# Functional analysis of polyketide synthase genes in the biocontrol fungus ***Clonostachys rosea***

**DOI:** 10.1038/s41598-018-33391-1

**Published:** 2018-10-09

**Authors:** Umma Fatema, Anders Broberg, Dan Funck Jensen, Magnus Karlsson, Mukesh Dubey

**Affiliations:** 1 Department of Forest Mycology and Plant Pathology, Uppsala Biocenter, Swedish University of Agricultural Sciences, P.O. Box 7026, SE-75007 Uppsala, Sweden; 2 Department of Molecular Sciences, Uppsala BioCenter, Swedish University of Agricultural Sciences, Box 7015, SE-75007 Uppsala, Sweden; 3 Present Address: Department of Plant and Soil Sciences, 412 Plant Science Building 1405 Veterans Drive, University of Kentucky, Lexington, KY 40546-0312 USA

## Abstract

*Clonostachys rosea* is a mycoparasitic fungus used for biological control of plant diseases. Its genome contains 31 genes putatively encoding for polyketide synthases (PKSs), 75% of which are arranged in biosynthetic gene clusters. Gene expression analysis during *C*. *rosea* interactions with the fungal plant pathogens *Botrytis cinerea* and *Fusarium graminearum* showed common and species-specific induction of PKS genes. Our data showed a culture media dependent correlation between PKS gene expression and degree of antagonism in *C*. *rosea*. The *pks22* and *pks29* genes were highly induced during fungal-fungal interactions but not during pigmentation, and gene deletion studies revealed that PKS29 was required for full antagonism against *B*. *cinerea*, and for biocontrol of fusarium foot rot on barley. Metabolite analysis revealed that Δ*pks29* strains has a 50% reduced production (*P* = 0.001) of an unknown polyketide with molecular formula C_15_H_28_O_3_, while Δ*pks22* strains lost the ability to produce four previously unknown polyketides named Clonorosein A-D. Clonorosein A and B were purified, their structures determined, and showed strong antifungal activity against *B*. *cinerea* and *F*. *graminearum*. These results show that PKS22 is required for production of antifungal polyketide Clonorosein A-D, and demonstrate the role of PKS29 in antagonism and biocontrol of fungal plant diseases.

## Introduction

Polyketides are a structurally diverse group of secondary metabolites with diverse functions important for ecological and evolutionary adaptation of fungi^1,2^. Polyketides are biosynthesised by large multi-domain enzymes called polyketide synthases (PKSs)^1,2^. Fungal PKSs are mainly iterative type I enzymes consisting of multiple functional domains: ketoacyl synthase (KS), acyl transferase (AT), dehydratase (DH), enoyl reductase (ER), ketoreductase (KR) and acyl carrier protein (ACP). PKS domains KS, AT and ACP catalyse the biosynthesis of acetyl coenzyme A and malonyl CoA and are essential domains, while KR, DH and ER are optional domains that may or may not be present in a single PKS protein. Based on the presence and absence of these optional domains, type I iterative PKSs can be functionally and phylogenetically grouped into highly reducing (HR) and non-reducing (NR) types, respectively. However, NR-PKSs contain additional unique functional S-acetyltransferase (SAT), product template (PT) and thioesterase (TE) domains^1,3,4^. Highly reducing PKS enzymes lacking KR, DH or ER are classified as partially-reducing (PR) PKSs. Variability in type, number, and activity of these domains contribute to the vast diversity of polyketide compounds produced by fungal PKSs.

Genes encoding PKSs are often localised in biosynthetic gene clusters (BGC) together with additional biosynthetic genes such as those encoding cytochrome P450 monooxygenases, oxidoreductases, dehydrogenases, acetyltransferases, methyltransferases, and other transferases responsible for modifications of the polyketide backbone to produce the end product. The BGC also includes genes encoding transporters like ATP-binding cassette (ABC) and major facilitator superfamily (MFS) transporters and regulatory proteins, for polyketide efflux and gene expression regulation, respectively^3,5^.

In fungi, functional characterization of PKSs have mostly been focussed on their role in human and plant pathogenesis and mycotoxin, drug and pigment production^1,2,6^. A role of polyketides in mycelial growth, and development of asexual and sexual structure has also been demonstrated^7,8^. However, only limited information is available concerning the role of polyketides in regulating microbial interactions, especially in interactions resulting in biological control of plant diseases. Fungal plant pathogen *Fusarium* spp. produce the polyketide mycotoxin zearalenone with high antifungal activity, presumably to compete with other fungi present in the same ecological niche^9^, and it was shown that the ability to detoxify zearalenone is indeed important for the biocontrol ability of the mycoparasitic fungus *Clonostachys rosea*^10,11^. Deletion of the PKS gene *pks4* in the weak mycoparasitic fungus *Trichoderma reesei* resulted in loss of pigmentation that made it more sensitive to toxic metabolites produced by the plant pathogenic fungi *Alternaria alternata*, *Botrytis cinerea*, *Rhizoctonia solani* and *Sclerotinia sclerotiorum*^12^.

The ascomycete fungus *C. rosea* (teleomorph name *Bionectria ochroleuca*)^13^ is an efficient biocontrol agent (BCA) against numerous plant pathogenic fungi^14–16^, against oomycete^17^ and plasmodiophorid^18^ plant pathogens and even against plant parasitic nematodes^19^. This broad activity against a wide taxonomic range of plant pathogens suggests that interference competition by means of secretion of broad spectrum antibiotic compounds may contribute to the biocontrol activity of *C. rosea*^20^. However, only a few peptaibols with antifungal activity^21^, epipolysulfanyldioxopiperazines with nematicidal activity^22^, polyterpenoid glisoprenins that inhibit appressorium formation of phytopathogenic fungi^23^, and a few TMC-151-type polyketide antibiotics with antibacterial properties^24^ have been identified from *C. rosea*.

This limited knowledge on production of secondary metabolites in *C. rosea* is in sharp contrast to the predicted gene content of its genome. The *C. rosea* strain IK726 genome contains high numbers of genes encoding PKSs (31 genes), non-ribosomal peptide synthetases (NRPSs, 17 genes), ABC (90 genes) and MFS (634 genes) membrane transporters^25,26^. Functional studies indeed confirmed the role of several ABC and MFS transporters in antagonism, xenobiotic tolerance and in biocontrol by mediating efflux of endogenous or exogenous metabolites in *C. rosea*^10,11,26–28^. However, the biological roles of PKSs in *C. rosea* are yet to be studied.

The aim of this study was to investigate the biological functions of PKSs in *C. rosea*, with emphasis on their role in biotic interactions with relevance for biological control of fungal plant diseases. We hypothesized that the significant copy number expansion of PKS genes is associated with the mycoparasitic lifestyle of *C. rosea*. To test this hypothesis, we identified PKS BGCs in *C. rosea*, and studied PKS gene expression patterns under different conditions relevant for mycoparasitic interactions and pigmentation. By generating and analysing gene deletion mutants, we identified previously unknown polyketides and investigated their biological functions. Our data demonstrate that PKSs are important for antagonism and mycoparasitic interactions in *C*. *rosea*. Moreover, our results further validate the potential to discover novel compounds by using a genome mining and reverse genetics approach.

## Results

### Identification and analysis of putative polyketide biosynthetic gene clusters

Analysis of the *C. rosea* IK726 genome with the antiSMASH software confirmed the presence of 31 PKS genes and one PKS-NRPS hybrid gene^25^ and predicted 20 putative PKS BGCs (18 BGCs with one PKS gene and two BGCs with two PKS genes; Tables 1 and S1). Conserved domain analysis using translated amino acid (aa) sequences verified the presence of PKSs genes in BGCs (Fig. S1). The predicted PKS BGCs ranged from 36.1 kbp to 59.6 kbp in size, contained 6–14 predicted genes, and were distributed to 20 different scaffolds (Table S1). Seven PKS genes (*pks1*, *pks5*, *pks11*, *pks14*, *pks15*, *pks17* and *pks26*) were not predicted to be part of a BGC, sequences of *pks30* and *pks31* were incomplete and *pks32* was a PKS-NRPS hybrid, and were thus excluded from further bioinformatic analyses. Analysis of the predicted BGC genes with the smCOG (secondary metabolite cluster of orthologous groups) and BlastP softwares showed the presence of a core polyketide biosynthetic gene, additional biosynthetic genes, regulatory genes and transport-related genes required for polyketide biosynthesis, backbone modifications, regulation and transport, respectively (Table 1). All PKS BGCs contained one to eight additional biosynthetic genes with the majority encoding putative flavoproteins such as flavin adenine dinucleotide (FAD)-binding domain proteins, flavin-containing monooxygenases and short-chain dehydrogenases/reductases (SDR). All PKS BGCs with the exception of clusters 63, 76, 93, 142 and 186, contained one or two genes encoding transport-related proteins with the majority being MFS transporters. In addition, more than 50% of the BGCs contained one or more genes encoding transcription factors (Table 1). A complete description of genes and their arrangement in the putative BGCs are presented in Table S2 and Fig. S2.

**Table 1:** Summary of the gene content in predicted PKS biosynthetic gene clusters^a^.

Cluster	PKS gene in cluster	Protein ID	Core biosynthetic genes^b^	Additional biosynthetic genes^c^	Transport-related genes^d^	Regulatory genes^e^
Cluster 9	*pks3*	BN869_T00000911_1	1	3	2	1
Cluster 49	*pks24*	BN869_T00005452_1	1	1	2	0
Cluster 52	*pks28*	BN869_T00005753_1	1	3	2	1
Cluster 63	*pks4*	BN869_T00006898_1	1	3	0	1
Cluster 67	*pks7* *pks9*	BN869_T00008884_1 BN869_T00006567_1	2	4	1	1
Cluster 76	*pks18*	BN869_T00007381_1	1	4	0	2
Cluster 78	*pks21*	BN869_T00007502_1	1	4	1	0
Cluster 93	*pks6*	BN869_T00008325_1	1	8	0	0
Cluster 113	*pks27*	BN869_T00009545_1	1	5	1	0
Cluster 117	*pks22*	BN869_T00009884_1	1	3	2	0
Cluster 122	*pks23*	BN869_T00010141_1	1	4	2	0
Cluster 124	*pks20*	BN869_T00010238_1	1	2	2	0
Cluster 130	*pks29*	BN869_T00010453_1	1	6	2	1
Cluster 134	*pks2* *pks12*	BN869_T00010693_1 BN869_T00010694_1	2	7	1	1
Cluster 142	*pks8*	BN869_T00011033_1	1	2	0	1
Cluster 168	*pks25*	BN869_T00012373_1	1	6	3	1
Cluster 180	*pks10*	BN869_T00013077_1	1	2	2	0
Cluster 186	*pks13*	BN869_T00013279_1	1	2	0	1
Cluster 188	*pks16*	BN869_T00013307_1	1	4	1	0
Cluster 190	*pks19*	BN869_T00013339_1	1	6	2	2

^a^A total of 22 predicted PKS genes were distributed to 20 putative PKS BGCs, while seven PKS genes were not localised to any BGSs (*pks1*, *pks5*, *pks11*, *pks14*, *pks15*, *pks17* and *pks26*).

^b^Genes encoding putative polyketide synthases.

^c^Genes encoding putative acetyltransferases, dehydrogenases, epimerases/dehydratases, hydrolases, isomerases, methyltransferases, oxidoreductases, transferases.

^d^Genes encoding putative ABC and MFS transporters.

^e^Genes encoding putative transcription factors.

**Figure 1:**
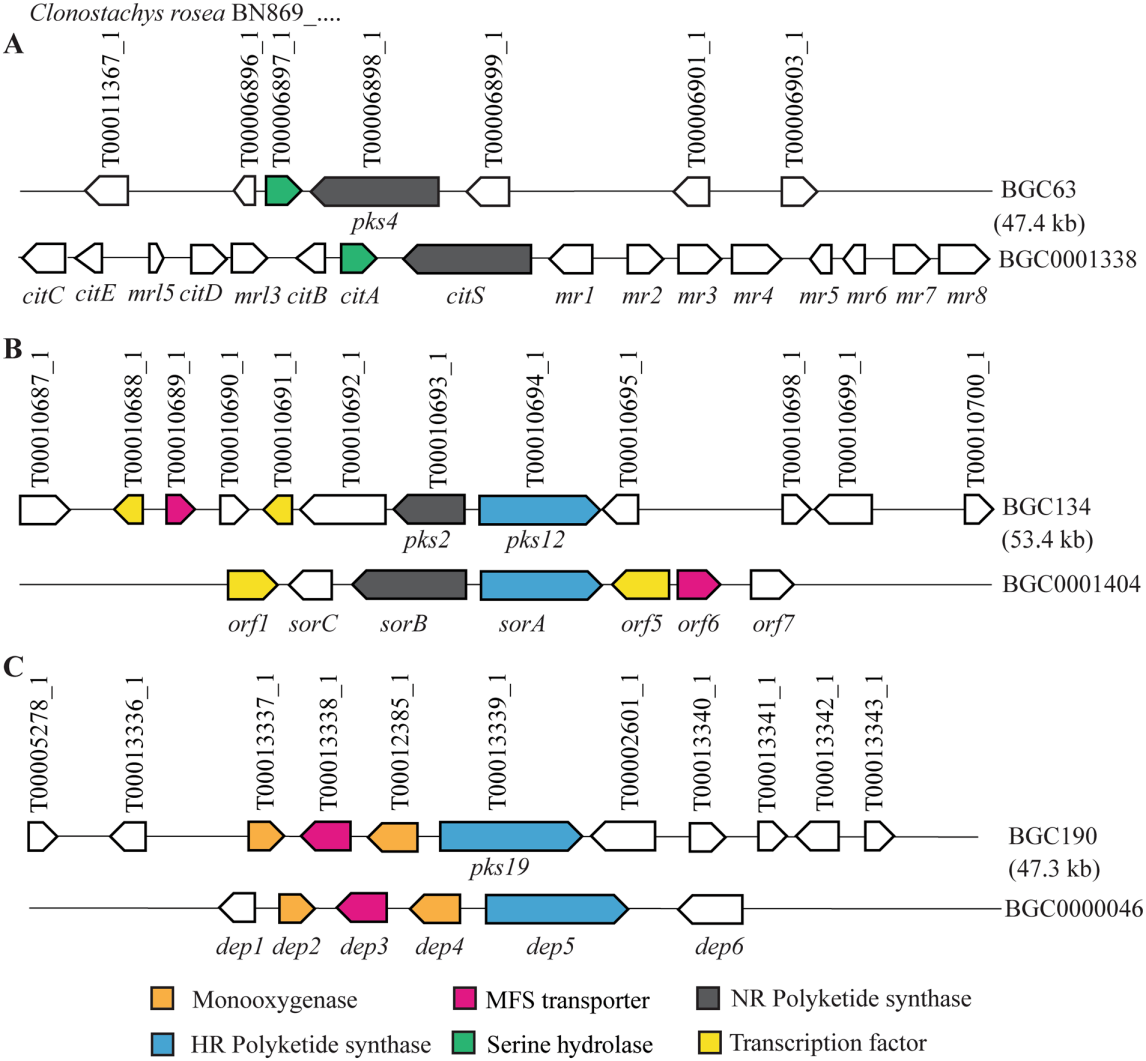
Schematic representation of *C. rosea* putative PKS biosynthetic gene clusters (BGCs) showing similarity with genes from characterised BGCs. (**A**) Citrinin BGC, (**B**) Sorbicillin BGC, and (**C**) Depudicin BGC. Type of genes common (≥40% sequence identity; e- value ≥1 × 10^25^) between two BGC are highlighted with the same colour, white arrows represent genes that are not common between BGC. Genes name are given in parentheses below the arrow, their orientation are indicated, while distance and size are not set to scale. *C. rosea* protein IDs are given above the arrows. Genbank accession numbers: *citA*: ALI92654.1; *citS*: ALI92655.1; *orf1*: CAP95402.1; *sorB*: CAP95404.1; *sorC*: CAP95405.1; *orf5*: CAP95406.1; *orf6*: CAP95407.1; *dep2*: ACZ57545.1; *dep3*: ACZ57546.1; *dep4*: ACZ57547.1; *dep5*: ACZ57548.1.

Cluster Blast analysis suggested that *C*. *rosea* PKS BGC63 was an ortholog of the characterised citrinin BGC (BGC0001338, Genbank accession number KT781075) of *Monascus ruber*^29^. The BGC63 contained seven genes from which a gene encoding a putative serine hydrolase and a gene encoding PKS4 showed ≥49% sequence identity with the serine hydrolase gene *CitA* (e-value = 1.8 × 10^−33^) and citrinin PKS gene *CitS* (e-value = 0) from the citrinin BGC BGC0001338. BGC134 showed similarity with the characterised sorbicillin BGC (BGC0001404, Genbank: AM920436) of *Penicillium chrysogenum*^30^. The BGC134 contained 12 genes including genes encoding two transcription factors, a MFS transporter, PKS2 and PKS12 that showed ≥55% sequence identity with genes encoding the transcription factors Orf1 (e-value = 2 × 10^−87^) and Orf5 (e-value = 1.8 × 10^−26^), the MFS transporter Orf6 (e-value = 8.4 × 10^−176^), and the two oppositely transcribed NR-PKS SorB (e-value = 5.6 × 10^−244^) and HR-PKS SorA (e-value = 0) from the sorbicillin BGC of *P*. *chrysogenum*, respectively. Four genes in BGC190 encoding two putative FAD-binding monooxygenases, a MFS transporter and PKS19 showed ≥44% sequence identity with the DEP2 (e-value = 3.4 × 10^−115^), DEP3 (e-value = 1.2 × 10^−68^), DEP4 (e-value = 1 × 10^−122^) and DEP5 (e-value = 0) genes in the depudecin BGC (BGC0000046, Genbank: FJ977165) of *Alternaria brassicicola*^31^ (Table S1, Fig. 1).

### Antagonistic effect of *Clonostachys rosea* is culture medium dependent

**Figure 2:**
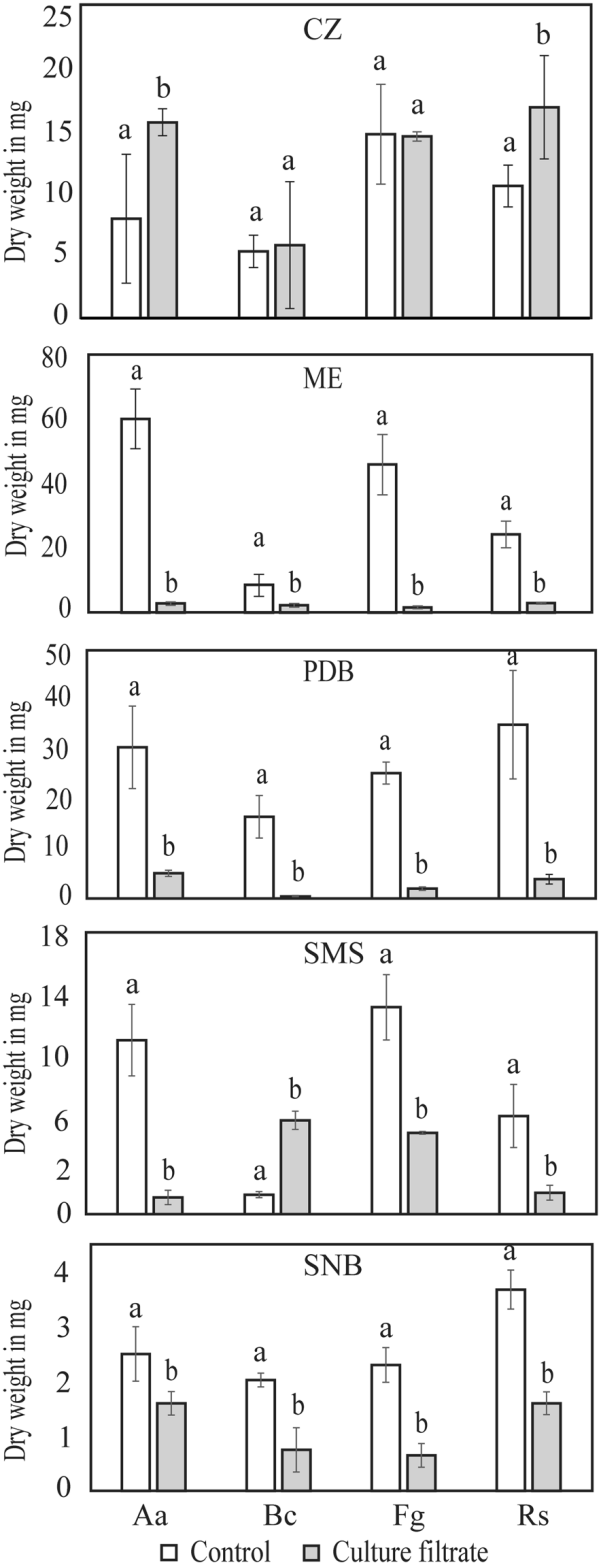
Production of fungal growth-inhibitory compounds by *C. rosea* in different media. *C. rosea* was grown in liquid medium broth (czapek-dox, CZ; malt extract, ME; potato dextrose, PDB; synthetic minimal salt, SMS; synthetic nutrient broth, SNB) for 4 days at 25 °C, culture filtrate was collected after removing the mycelium, and was then inoculated with an *A*. *alternata* (Aa), *B*. *cinerea* (Bc), *F*. *graminearum* (Fg) or *R*. *solani* (Rs) agar plug. Fungus inoculated into the respective fresh culture broth was used as control. Biomass production in culture filtrates was analysed by determining mycelial dry weight 4 days post-inoculation. Error bars indicate standard deviation based on five biological replicates. Different letters indicate statistically significant differences (*P* ≤ 0.05) within experiments based on the Fisher’s exact test.

Antagonistic ability of *C. rosea* against the plant pathogenic fungi *A*. *alternata*, *B*. *cinerea*, *Fusarium graminearum*, and *R*. *solani* was determined by measuring their mycelial biomass in *C. rosea* culture filtrates from culture media differing with regard to carbon source, nitrogen source and pH. The media compositions are provided in Table S3. The mycelial biomass of all plant pathogenic fungi was reduced significantly (*P* ≤ 0.05) in *C. rosea* culture filtrates from all culture media, except Czapek-Dox (CZ), in comparison to control medium (Fig. 2). The maximum reduction in biomass was observed in *C. rosea* culture filtrates from potato dextrose broth (PDB) (83–98%) and malt extract (ME) (73–97%) followed by synthetic minimal salt (SMS) (61%–90%) and synthetic nutrient broth (SNB) (36–72%). Interestingly, *A*. *alternata* and *R*. *solani* produced significantly more biomass (*P* ≤ 0.030) in *C. rosea* culture filtrate from CZ medium, compared with the CZ control (Fig. 2). We observed that *C. rosea* lowered the pH in CZ medium from 7.2 to 5.5 during incubation, and the result from a follow-up experiment showed that *A. alternata* and *R. solani* both produced significantly (*P* ≤ 0.01) more biomass in CZ medium with pH 5.5 as compared with CZ medium with pH 7.2 (data not shown).

### Gene expression analysis

Gene expression analysis of the 31 PKS genes and one PKS-NRPS hybrid gene in *C. rosea* was carried out in 3 different conditions: (i) during mycelial growth in liquid culture media, (ii) during dual culture interactions with plant pathogenic fungal species *B*. *cinerea* or *F*. *graminearum*, and (iii) during pigment production on solid agar.

**Figure 3:**
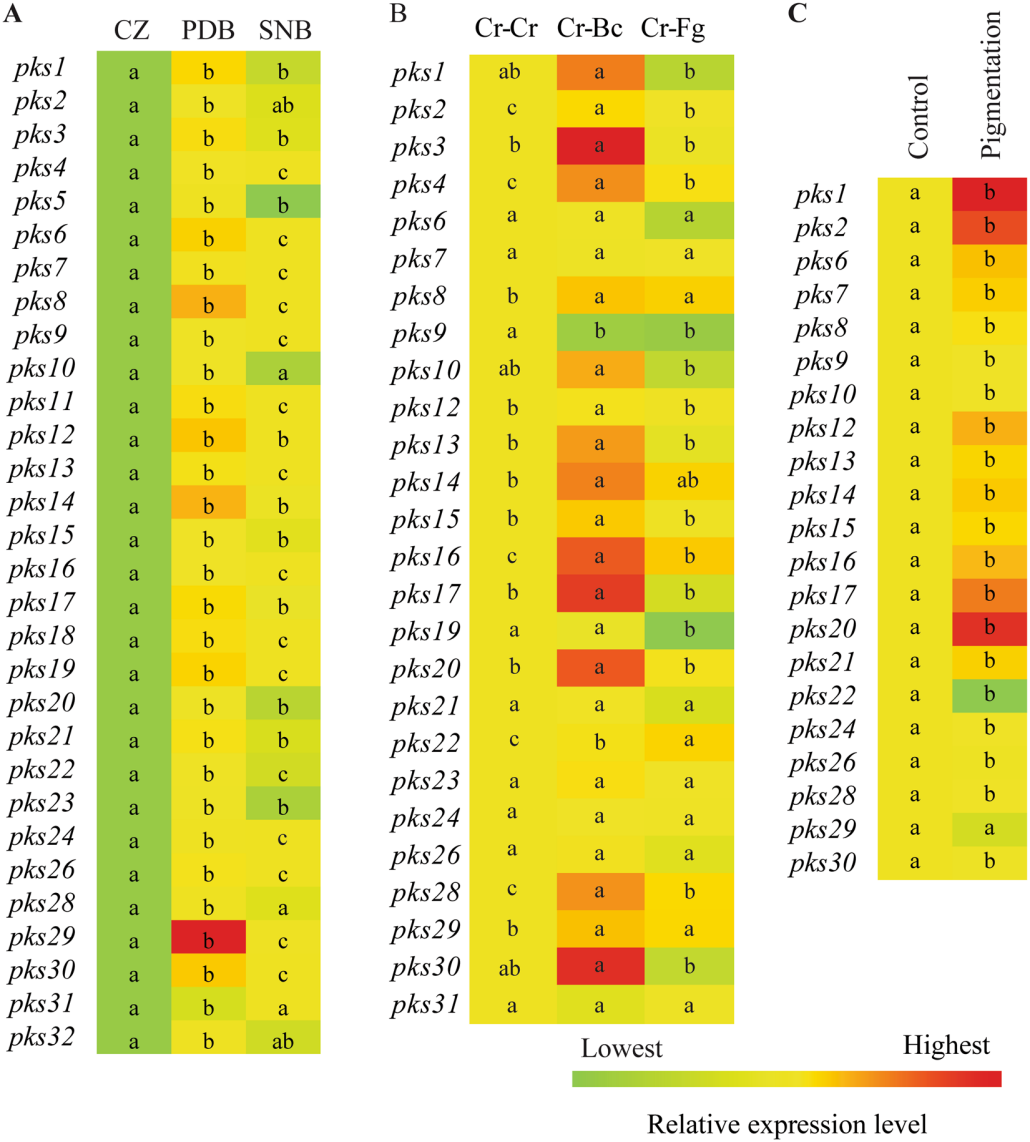
Heat map of polyketide synthase gene expression in *C. rosea*. (**A**) During mycelial growth in liquid CZ, PDB and SNB culture media. *C. rosea* mycelia grown in CZ medium were used as control treatment. (**B**) During interactions with itself (Cr-Cr), *B*. *cinerea* (Cr-Bc) or *F*. g*raminearum* (Cr-Fg). *C*. *rosea* interaction with self (Cr–Cr) was used as control treatment. (**C**) During pigment production. Four days old *C. rosea* culture plates, incubated at the same conditions were used as control treatment as no pigmentation was observed at this stage. Relative expression level based on RT-qPCR was calculated as the ratio between the target PKS gene and β-tubulin using 2^−ΔΔCt^ method (Livak and Schmittgen, 2001), and compared with the respective control. Statistically significant differences (*P* ≤ 0.05) in gene expression between treatments were determined using Fisher’s exact test and are indicated by different letters. The scale representing the relative expression intensity values is shown.

In order to investigate if there was a correlation between degrees of antagonism in different culture media with PKS gene expression, CZ (no significant positive antagonistic effect), PDB (high antagonistic effect) and SNB (medium antagonistic effect) media were selected for PKS gene expression analysis. With the exception for *pks25* and *pks27*, all PKS genes were expressed in the three selected culture media (Fig. 3A). The expression levels of all genes were significantly (*P* ≤ 0.002) higher in PDB compared with CZ. Similarly, 24 PKS genes showed significantly (*P* ≤ 0.002) induced expression in SNB compared with CZ. Eighteen genes were induced significantly (*P* ≤ 0.002) in PDB compared with SNB (Fig. 3A). In PDB, *pks29* showed the maximum 3154-fold induction followed by *pks8* (926-fold), *pks14* (892-fold), *pks12* (660-fold), *pks3*0 (640-fold), *pks6* (526-fold) and *pks19* (506-fold). In SNB, *pks29* showed maximum 35-fold induction followed by *pks16* (16-fold), *pks4*, *pks9*, *pks24* (13-fold), and *pks12* (12-fold). Interestingly, no induction in gene expression was observed in *C*. *rosea* grown in CZ compared with PDB or SNB (Fig. 3A).

During fungal interactions, a common set of 7 PKS genes were significantly (*P* ≤ 0.020) induced during interactions with both *B. cinerea* (Cr-Bc) and *F. graminearum* (Cr-Fg) as compared with the self-interaction control (Cr-Cr) (Fig. 3B), although with moderate fold changes (maximum 4.3-fold induction). Among these, four genes (*pks2*, *pks4*, *pks16*, and *pks28*) were more highly expressed in Cr-Bc than Cr-Fg, while *pks22* showed the opposite pattern with higher expression in Cr-Fg. Another 7 PKS genes (*pks3*, *pks12*, *pks13*, *pks14*, *pks15*, *pks17* and *pks20*) were specifically induced 1.4–6.2 fold (*P* ≤ 0.049) against *B. cinerea* but not *F. graminearum*. The expression of *pks9* was significantly suppressed (*P* = 0.001) in both Cr-Bc and Cr-Fg, while *pks19* was significantly suppressed (*P* = 0.001) exclusively during interaction with *F. graminearum* (Fig. 3B). Seven PKS genes (*pks6*, *pks7*, *pks21*, *pks23*, *pks24*, *pks26*, *pks31*) were expressed but showed no significant changes in expression during interactions, while transcripts from another 6 PKS genes (*pks5*, *pks11*, *pks18*, *pks25*, *pks27* and *pks32*) were not detected under these conditions (Fig. 3B).

*C. rosea* often secretes yellow pigment during cultivation on PDA. To study the role of PKSs in ageing and yellow pigment biosynthesis, PKS gene expression was analysed at the time of occurrence of yellow pigment on agar plates (10 days post-inoculation [dpi]) and compared with an earlier time point (4 dpi) where no pigmentation was evident. Gene expression analysis showed a significant (*P* ≤ 0.011) induction in expression of 19 PKS genes, significant suppression (*P* = 0.047) of *pks22*, and no change in expression of *pks29* (*P* = 0.764) during pigmentation compared with the control (Fig. 3C). The PKS gene *pks1* showed the maximum 933-fold induction followed by *pks20* (797-fold), *pks2* (646-fold), and *pks17* (464-fold). Expression of 11 PKS genes (*pks3, pks4, pks5*, *pks11*, *pks18*, *pks19*, *pks23*, *pks25*, *pks27*, *pks31*, and *pks32*) was not detected under these conditions (Fig. 3C).

### Sequence analysis of PKS22 and PKS29

Due to their high induction during fungal-fungal interactions but no induction during pigmentation, *pks22* and *pks29* were selected for functional characterization, as the best candidates for biosynthesis of polyketides involved in microbial interactions. The *pks22* and *pks29* open reading frames (ORFs) were predicted to encode polypeptides composed of 2490 and 2378 aa residues, respectively. The conserved domain analyses of PKS22 and PKS29 predicted the presence of functional domains KS, AT, DH, ER, KR and ACP similar to the typical HR type polyketide synthase domains, and an additional methyltransferase (MT) domain (Fig. S1), which is in line with previous results from a phylogenetic analysis that placed PKS22 and PKS29 in a HR polyketide group^25^. The antiSMASH analysis showed that *pks22* was a core biosynthetic gene clustered with additional biosynthetic genes putatively encoding an aldo/keto reductase, a tubulin-tyrosine ligase, a FAD-dependent oxidoreductase, an AMP-binding protein and a MFS transporter in the 48.7 kbp long BGC117. Similarly, *pks29* was a core biosynthetic gene clustered with additional biosynthetic genes putatively encoding a FAD-dependent oxidoreductase, an AMP-dependent synthetase and ligase, a short-chain dehydrogenase/reductase, a copper type II ascorbate-dependent monooxygenase, a cytochrome b561, a flavin-containing amine oxidoreductase, a tryptophan halogenase, two MFS transporters and a transcription factor in the 54.2 kb long BGC130 (Table 1, Fig. S2). BlastP analysis using the PKS22 aa sequence showed highest similarity (49% identity) with PKSs from *Rosellinia necatrix* (GAP88125) and *Podospora anserina* (CDP29160) followed by 44% identity with a lovastatin nonaketide synthase from *Escovopsis weberi* (KOS22576). The PKS29 aa sequence showed highest similarity (53% identity) with the same PKS from *R. necatrix* (GAP88125) as PKS22, followed by 50% identity with the lovastatin diketide synthase LovF from *Madurella mycetomatis* (KXX72797) and 43% identity with a PKS from *Glarea lozoyensis* (XP_008078275) known to be involved in biosynthesis of the antifungal lipohexapeptide pneumocandin^32^.

### Generation and validation of gene deletion mutants

Gene deletion mutants of *pks22* and *pks29* were generated by replacing the respective orf with the hygB selection cassette by homologous recombination using *Agrobacterium tumefaciens-*mediated transformation (ATMT). More than 75 hygromycin resistant *C. rosea* colonies were obtained for each transformation on selection plates containing hygromycin (200 μg/ml) and cefotaxime (300 μg/ml). The individual transformants were sub-cultured on fresh selection plates and were subjected for mutant validation to confirm that the deletion cassette was inserted at the target locus using PCR with primers located within the hygB cassette together with primers located upstream and downstream of the construct (Fig. S3A) as described in our previous studies^11,33^. The PCR fragments of expected size were amplified in five and four transformants for *pks22* and *pks29* deletion mutants, respectively, while no amplification was observed in wild type (WT) (Fig. S3B,C), indicating that *pks22* or *pks29* were replaced correctly by hygB in these transformants. Furthermore, RT-PCR experiments on cDNA using primers specific to the *pks22* or *pks29* sequence demonstrated the complete loss of *pks22* or *pks29* transcript in each transformant, whereas an amplification product of desired size was found in WT strain (Fig. S3B,C). In order to confirm that the observed phenotypes were attributed to deletion of *pks22* or *pks29* and not to ectopic insertions, if any, of the deletion cassette, all 5 independent deletion strains of *pks22* (Δ*pks22*A, Δ*pks22*B, Δ*pks22*C, Δ*pks22*D, Δ*pks22*E), and 4 independent deletion strains of *pks29* (Δ*pks29*A, Δ*pks29*B, Δ*pks29*C, Δ*pks29*D) were used in phenotypic analyses, unless otherwise specified.

### Deletion of PKS genes resulted in phenotypic effects

**Figure 4:**
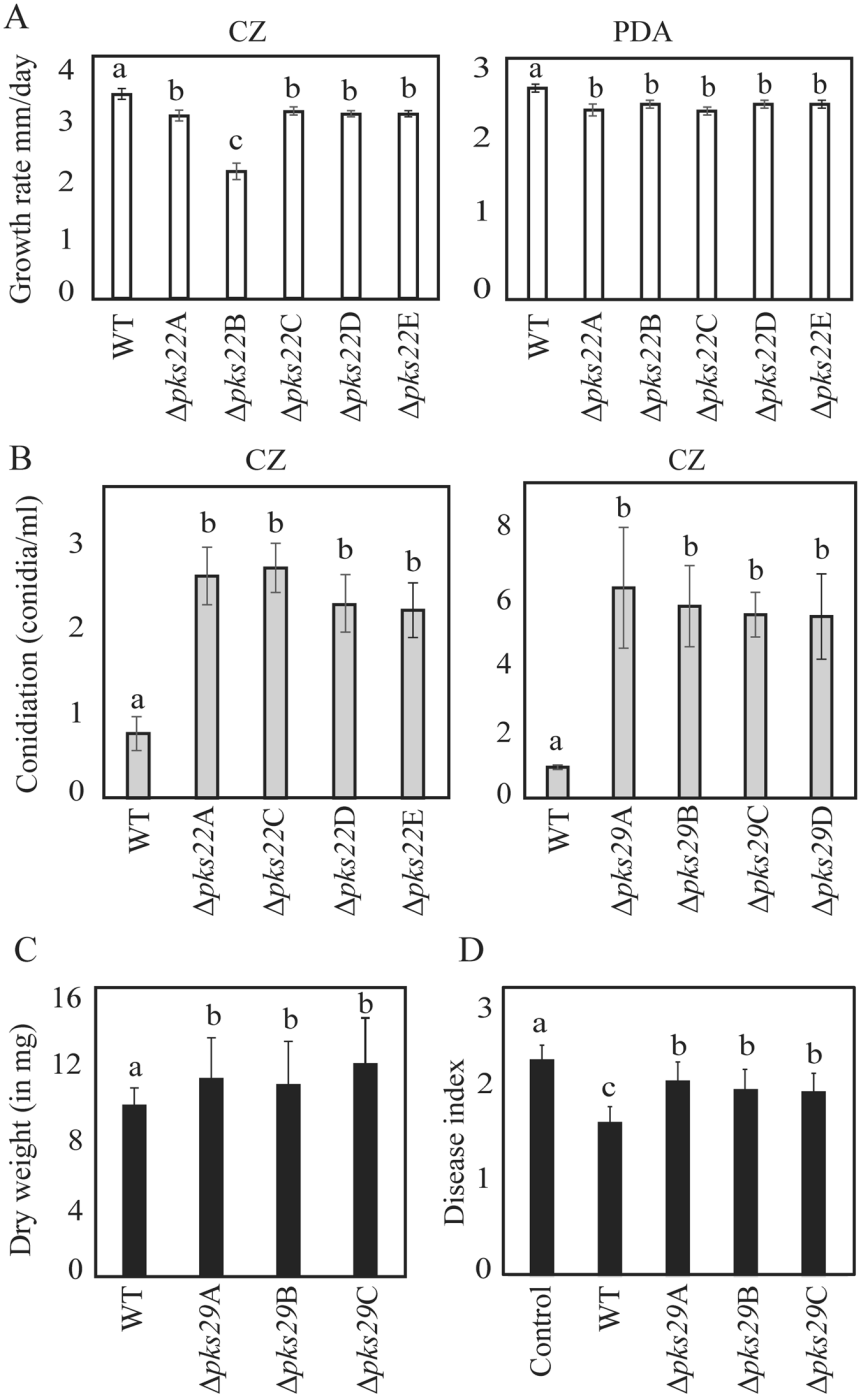
Phenotypic characterizations of *C*. *rosea* WT and deletion mutants. (**A**) Growth rate of WT, and *pks22* deletion strains on czapek-dox (CZ) or potato dextrose agar (PDA) medium. Strains were inoculated on solid agar medium, incubated at 25 °C and the growth rate was recorded five days post-inoculation. Error bars represent standard deviation based on 4 biological replicates. (**B**) Conidiation of WT, *pks22* and *pks29* deletion strains on CZ medium 12 days post-inoculation. Conidia were harvested in equal volume of water and counted using a Bright-Line Haemocytometer as per instruction of manufacturer. Error bars represent standard deviation based on 4 biological replicates. (**C**) Culture filtrate test of *C. rosea* strains. Culture filtrates from WT and deletion strains grown in PDB were collected 10 days post-inoculation and then inoculated with a *B. cinerea* agar plug. Biomass production in culture filtrates was analysed by determining mycelial dry weight 4 days post-inoculation. Error bars represent standard deviation based on 4 biological replicates. (**D**) *In vivo* bioassay to test the biocontrol ability of *C*. *rosea* strains against *F. graminearum* foot rot disease on barley. Barley seeds were coated with *C. rosea* conidia, and planted in moist sand together with a *F. graminearum* agar plug. Seedlings were harvested three weeks post-inoculation and disease symptoms were scored on 0–4 scale. The experiment was performed in five biological replicates with 12–15 plants in each replicate. Different letters indicate statistically significant differences (*P* ≤ 0.05) within the experiments based on the Fisher’s exact test.

A significant (*P* ≤ 0.004) reduction in mycelial growth of Δ*pks22* strains compared with WT was recorded on PDA and CZ-agar medium (Fig. 4A). However, deletion of *pks29* showed no effect on mycelial growth rate (data not shown). Deletion strain Δ*pks22*B showed a significantly lower growth rate compared with the other 4 independent Δ*pks22* strains, and was thus excluded from further phenotypic analyses. Furthermore, the Δ*pks22* and Δ*pks29* strains showed significantly (*P* ≤ 0.001) increased conidiation on CZ-agar (Fig. 4B). However, no difference in conidiation was observed between WT and deletion strains on PDB medium (data not shown). Dual culture interaction assays showed no significant differences in antagonistic ability between WT and *pks22* or *pks29* deletion strains against the plant pathogenic fungi *A*. *altarnata*, *B*. *cinerea*, *F*. *graminearum* or *R*. *solani*. Dual culture interactions were allowed up to 20 days and no differences in overgrowth on the prey fungi were observed between WT and deletion strains. Furthermore, a culture filtrate test was performed to assess the difference in antagonism by measuring mycelial biomass of *B*. *cinerea* or *F*. *graminearum* in culture filtrates of *C*. *rosea* WT and deletion strains. Similar to the dual culture interaction test on agar plates, no significant differences in biomass of *B*. *cinerea* or *F*. *graminearum* when grown in WT or Δ*pks22* liquid culture filtrates were identified (data not shown). However, a significant (*P* ≤ 0.024) increase in biomass of *B*. *cinerea* grown in PDB culture filtrates obtained from Δ*pks29* strains was found in comparison to biomass produced in WT culture filtrates (Fig. 4C), while the similar experiment against *F*. *graminearum* showed no significant differences in biomass production (data not shown). In contrast with the *in vitro* antagonism tests, a bioassay for *F*. *graminearum* foot rot disease on barley showed a significant (*P* ≤ 0.027) increase of disease severity in barley seedlings previously seed coated with Δ*pks29* strains as compared with seedlings from seeds coated with WT *C. rosea* (Fig. 4D). However, disease symptoms on seedlings from seeds coated with Δ*pks22* strains showed no significant difference compared with WT.

### Secondary metabolite analyses led to the identification of novel compounds

**Figure 5:**
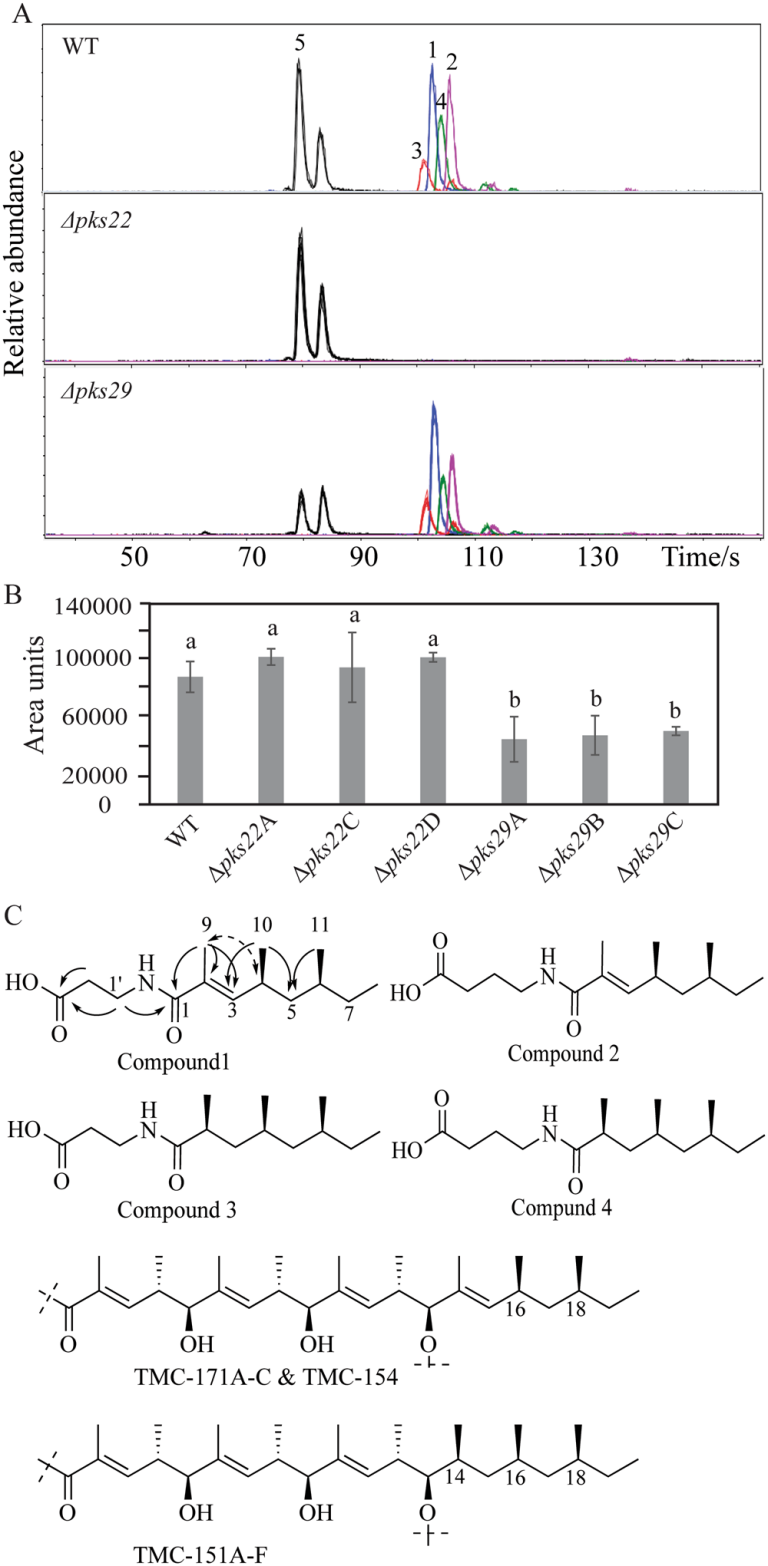
Secondary metabolite analysis of *C*. *rosea* WT and deletion strains. (**A**) Extracted ion chromatograms of compounds **1**–**5** obtained by UHPLC-MS analysis of WT, and deletion strains culture filtrates. The experiment was performed in three biological replicates. (**B**) Relative quantification of compounds **5** in culture filtrates of *C*. *rosea* WT and deletion strains. Quantification was done by UHPLC-MS using representative extracted-ion chromatograms for the compound. Sample areas are presented as relative area units. Error bars represent standard deviation based on three biological replicates. (**C**) Proposed structures of compounds **1**–**4**, and key HMBC (solid arrows) and ROESY (dashed arrow) correlations for structure determination of compound 1, along with partial structures of TMC-171A-C, TMC-154, and TMC-151A-F (Kohno *et al*.^34^; Kohno *et al*.^35^).

In order to investigate the involvement of PKS22 or PKS29 in secondary metabolite production, culture filtrates of *C*. *rosea* strains were analyzed by ultra-high-performance liquid chromatography mass spectrometry (UHPLC-MS). Our data showed that four metabolites detected at the retention time of 90–98 seconds (s) with m/z values (H^+^) 256.1907 (compound **1**), 270.2063 (compound **2**), 258.2057 (compound **3**), and 272.2222 (compound **4**) in samples from WT and Δ*pks29* strains were not detected from Δ*pks22* strains, suggesting that the Δ*pks22* strains lost the ability to produce compounds **1**–**4** (Fig. 5A). In addition, UHPLC-MS analysis showed a significant (*P* = 0.001) 50% reduction of a compound eluted at around 70 s retention time with m/z (Na^+^) 279.1934 (compound **5**) in culture filtrates from Δ*pks29* strains compared with WT and Δ*pks22* strains (Fig. 5A,B).

According to the mass spectrometry (MS) data, compounds **1–4** had molecular formulae C_14_H_25_NO_3_, C_15_H_27_NO_3_, C_14_H_27_NO_3_, and C_15_H_29_NO_3_, respectively, with unsaturation index three, three, two and two, respectively. Compounds **1** and **2** were isolated by preparative high-performance liquid chromatography (HPLC) (0.25 mg and 0.32 mg, respectively), and their structures were determined by nuclear magnetic resonance (NMR) (Table 2), MS and MS/MS. Using correlation spectroscopy (COSY) and total correlation spectroscopy (TOCSY) NMR experiments, two spin-systems, A and B, were identified in compound **1**. Spin-system A comprised two adjacent methylene groups and a heteroatom linked hydrogen, possibly an amide proton, i.e. tentatively -CH_2_-CH_2_-NH-CO-. Spin-system B contained signals from three methyl groups (one sp^2^ linked, two methine linked, and one methylene linked), one sp^2^ linked hydrogen, two methine groups and two methylene groups, joined to form a CH_3_-CH_2_-CH(CH_3_)-CH_2_-CH(CH_3_)-CH=C-CH_3_ motif. Spin-systems A and B were shown to be connected by heteronuclear multiple bond correlation (HMBC) experiments, by a cross-peak from the sp^2^ linked methyl group of spin-system B to a carbonyl at δ_C_ 169.7, which also had a cross-peak to the methylene group in the proposed amide-end of spin-system A (Fig. 5C). After comparison with the molecular formula C_14_H_25_NO_3_, it was deduced that a COOH group also should be included in the structure, which only could be placed on the other end of spin-system A. This was supported by HMBC cross-peaks from the two methylene groups of spin-system A to a carbonyl carbon at δ_C_ 173.8. The C-2/C-3 double bond was determined to have E-configuration by a rotating-frame Overhauser spectroscopy (ROESY) cross-peak between H_3_-9 and H-4, resulting in the proposed structure of compound 1 (Fig. 5C), for which we propose the name Clonorosein A. The structure for 1 was supported by MS/MS analysis, which yielded one major fragment ion with m/z 167.1426, in agreement with an acylium ion formed by cleavage of the proposed amide bond. The NMR data for compound 2 was very similar to the data for **1**, the only major difference was the presence of one extra methylene group in spin-system A, i.e. -CH_2_-CH_2_-CH_2_-NH-CO-, resulting in the proposed structure for the compound **2** (Fig. 5C), which was named Clonorosein B. Compounds **1** and **2** both showed positive specific rotation (+48 and +33, respectively), but the absolute configuration was not determined experimentally for the compounds. Clonorosein A and B are both new structures, but the methylated octenoyl moiety of A and B is present in reduced form in the compounds TMC-171A, B, C, and TMC-154^34,35^ (Fig. 5C). These compounds were isolated from different strains of *C. rosea* (formerly *Gliocladium roseum*/*G. catenulatum*) TC 1304 and TC 1282 and it is likely that Clonorosein A and B share the configuration at C-4 and C-6 with the configuration at C-16 and C-18 in TMC-171A, B, and C, which was determined to be S^34^. Thus, the 4S, 6S configuration was proposed for Clonorosein A and B.

**Table 2:** ^1^H and ^13^C NMR data for compound **1** and **2** (600 MHz and 150 MHz, respectively, CDCl_3_, 30 °C).

Positions.	Compound 1		Compound 2	
	^13^C	^1^H	^13^C	^1^H
1	169.9	—	170.8	—
2	128.7	—	128.5	—
3	143.4	6.13 (d, 10.0 Hz)	143.6	6.17 (d, 10.0 Hz)
4	30.6	2.59 (m)	30.7	2.60 (m)
5	44.3	1.33 (m)	44.4	1.35 (m)
		1.13 (m)		1.14 (m)
6	32.4	1.25 (m)	32.4	1.26 (m)
7	30.1	1.29 (m)	30.0	1.31 (m)
		1.11 (m)		1.15 (m)
8	11.4	0.86 (t, 7.3 Hz)	11.4	0.86 (t, 7.3 Hz)
9	12.8	1.86 (s)	12.7	1.88 (s)
10	20.9	0.98 (d, 6.7 Hz)	21.0	0.99 (d, 6.7 Hz)
11	19.4	0.83 (d, 6.4 Hz)	19.3	0.84 (d, 6.4 Hz)
NH	—	6.31 (m)	—	6.02 (m)
1′	35.1	3.61 (q, 5.6 Hz)	39.1	3.44 (q, 4.3 Hz)
2′	33.9	2.68 (t, 5.8 Hz)	25.8	1.92 (m, 6.5 Hz)
3′	173.8	—	31.7	2.44 (dd, 6.7, 7.4 Hz)
4′	—	—	174.3	—

Compounds **3** and **4** were proposed, by the respective molecular formulae, to differ from Clonorosein A and B, by the presence of two extra hydrogen atoms (Fig. 5C). Compounds **3** and **4** were not isolated in sufficient amounts or purity for allowing detailed analysis by NMR, but for both compounds the signals from the olefinic H-3 and the sp^2^ linked methyl group were absent, indicating that the difference between Clonorosein A and compound **3**, and between Clonorosein B and compound **4**, was the absence of the C-2/C-3 double bond in the fatty acyl part of the molecules. This was also in accordance with MS/MS data. In analogy with the configuration of C-4 and C-6 in compounds **1** and **2**, C-2, C-4 and C-6 in compounds **3** and **4** were all tentatively assigned to have S-configuration after comparison with the compounds TMC-151A-F produced by *C. rosea* TC 1280^35^. Compounds **3** and **4** were given the names Clonorosein C and D, respectively.

The molecular formula of compound **5**, C_15_H_28_O_3_, obtained by MS, corresponded to an unsaturation/ring index of two, and could fit many compounds including e.g. a monounsaturated hydroxy substituted fatty acid. Unfortunately, we were not able to isolate compound **5** of sufficient quantity or purity for structure determination by NMR. A similar compound with the same m/z was eluted at 74 s (Fig. 5A), and the mass spectrum and MS/MS data for this compound was very similar to the data for the compound eluting at 70 s. These two compounds are thus presumably of similar structures, and may differ in e.g. relative configuration or perhaps position of substituents.

### Clonorosein A and Clonorosein B exhibited antifungal activity

**Figure 6:**
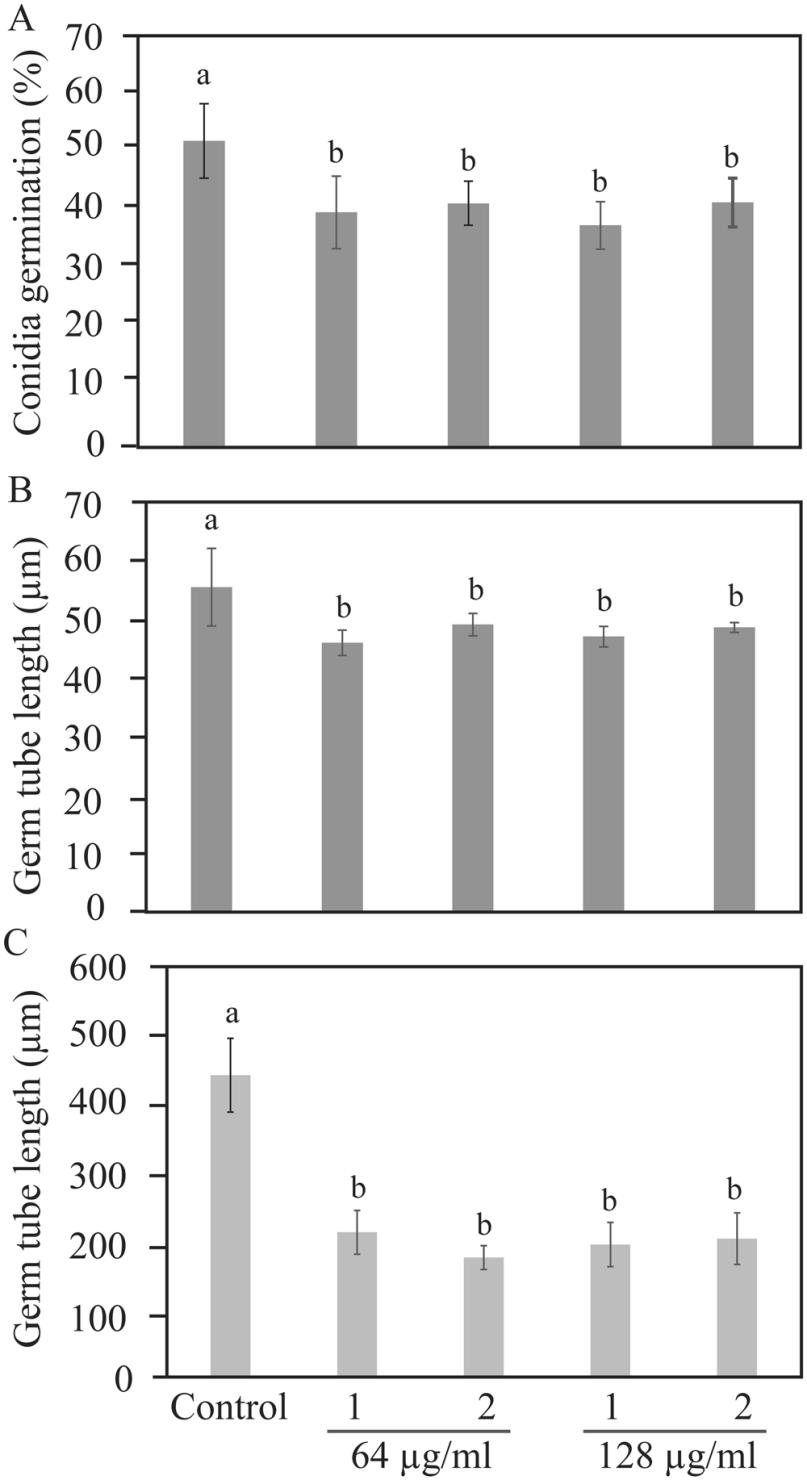
Antifungal activity of compound **1** and compound **2** extracted from *C*. *rosea* against plant pathogens *B*. *cinerea* and *F*. g*raminearum*. Conidial suspensions in half-strength potato dextrose broth were inoculated in 96-well microtiter plates containing compound **1** or compound **2**. Frequency of germinating conidia was determined 8 hour post-inoculation by counting the number of germinating and non-germinating conidia, while germ tube length was measured using ImageJ software. (**A**) *B*. *cinerea* conidia germination, (**B**) *B*. *cinerea* germ tube length, (**C**) *F*. *graminearum* germ tube length. Abbreviation: 1, compound **1**; 2, compound **2**. Concentrations of compound are indicated below the bar. Error bars represent standard deviation based on three biological replicates. Different letters indicate statistically significant differences (*P* ≤ 0.05) within the experiments based on Fisher’s exact test.

Clonorosein A and B isolated and purified from *C. rosea* culture filtrates were used to test their antifungal activity by analyzing conidial germination and germ tube development of *B*. *cinerea* and *F*. *graminearum*. Both compounds significantly inhibited conidial germination (*P* ≤ 0.028) and germ tube growth (*P* ≤ 0.042) of *B*. *cinerea* at a concentration of 64 µg/ml compared with the control (Fig. 6A,B). The two compounds showed an even more severe effect against *F*. *graminearum* with a >50% (*P* = 0.001) reduction in conidial germ tube length already at 64 µg/ml compared with the control (Fig. 6C). However, no significant difference was found in *F*. *graminearum* conidial germination at any tested concentration (data not shown). The antifungal effects of Clonorosein A and B did not appear to be dose-dependent, as no significant differences were observed between the concentrations 64 and 128 µg/ml.

## Discussion

As an opportunistic, necrotrophic mycoparasite with broad host range, *C*. *rosea* has evolved strategies to compete with and antagonise other fungi by producing bioactive secondary metabolites^36^. This contributes to its usefulness as a biological control agent to protect crop plants against fungal and other diseases in agricultural production systems^20^. This ability for antibiosis is reflected by the presence of a rich repertoire of genes encoding enzymes associated with secondary metabolite production in the *C. rosea* genome, including 31 PKSs, one PKS-NRPS hybrid, 17 NRPSs, and 8 terpene synthases^25^. An evolutionary analysis showed selection for increased PKS gene copy number in *C*. *rosea* compared with other hypocrealean fungi such as the mycoparasitic *T*. *atroviride* and *T*. *virens* (18 putative PKS genes), saprotrophic *T*. *reesei* (11 putative PKS genes) and plant pathogenic *F*. *graminearum*, *F*. *oxysporum* f. sp. *lycopersici*, *F*. *solani* or *F*. *verticillioides* (12–16 PKS genes)^25^. Our work shows that most PKS genes in *C*. *rosea* (75%) are located in predicted secondary metabolite biosynthetic clusters, which is a higher proportion compared with the mentioned *Trichoderma* spp. (50%)^37^, indicating that the increase of PKS gene copy number in *C*. *rosea* has specifically involved genes related with secondary metabolite production.

Our gene expression data further suggest that antagonism is an important driving force behind the selection for increased PKS gene copy number in *C. rosea*. First, this is supported by the positive correlation between fungal growth inhibitory activity of different culture filtrates and the number and fold-change of expressed PKS genes, with PDB exhibiting the highest growth inhibiting activity and the highest number of expressed PKS genes. The fact that all tested fungal plant pathogens, *A. alternata*, *B. cinerea*, *F. graminearum* and *R. solani*, displayed reduced growth in *C. rosea* PDB, ME, SMS and SNB culture filtrates suggests production of compounds with broad spectrum activity, or production of a mix of compounds with different biotic activities. The exact identity of the growth media constituents that trigger PKS gene expression in *C. rosea* is not known, but it is reported that the source and level of carbon and nitrogen, and pH can influence production of secondary metabolites in fungi^38^. In contrast, lack of growth inhibitory activity towards any fungus in CZ culture filtrate may suggest that the higher pH (7.3 ± 0.2) and nitrogen (2% NaNO3), and sucrose as the sole carbon source (3% sucrose) in CZ may not represent optimal conditions for PKS gene expression and consequently secondary metabolite production in *C*. *rosea*. In fact, *C. rosea* lowered the pH in CZ medium from 7.2 to 5.5 during incubation, which may explain the increased growth of *A. alternata* and *R. solani* in the culture filtrate compared with the CZ control. This result is in line with previous data from *F. graminearum* where higher number of PKS genes were expressed in PDB medium, rice agar and corn meal medium compared with CZ medium^7^. Similar results are reported previously from the functional analysis of *bik* genes responsible for the biosynthesis of the red pigment bikaverin in *F*. *fujikuroi*^39,40^. Expression of *bik* genes was highly suppressed at neutral and alkaline pH, and at high nitrogen concentration, while their expression was increased at acidic pH and lower nitrogen concentration. The nitrogen and pH-mediated regulation of gene expression was consistent with the production of the bikaverin^39,40^.

Secondly, 45% of all PKS genes in *C. rosea* are induced in response to antagonistic interactions with *B*. *cinerea* or *F*. *graminearum*. PKS genes are also reported to be induced in *T*. *atroviride*, *T*. *reesei* and *T*. *harzianum* during interaction with *R. solani* and *Sclerotinia sclerotiorum*, respectively^41,42^. Data from *C*. *rosea* interacting with *B. cinerea* or *F. graminearum* revealed both common and species-specific responses in PKS gene expression. This is in line with a recent transcriptome analysis of *C. rosea* during interactions with *B. cinerea* or *F. graminearum* where genes predicted to encode proteins particularly involved in membrane transport and secondary metabolite biosynthesis were differentially expressed towards the two fungi^26^. The *pks9* gene was reported by Nygren *et al*.^26^ to be induced specifically against *B. cinerea*, while it is repressed during interactions with both fungi in the current work. One important difference between these studies that may explain this contradiction is the fact that Nygren *et al*.^26^ sampled the hyphal front of *C. rosea* 24 hours after contact with the antagonists, while samples were taken at contact (24 hours earlier) in the current work.

It is possible that specificity in expression of PKS genes towards different fungal prey species represents an adaptation of the mycoparasitic attack driven by intrinsic differences between the fungal preys. Alternatively, specificity in *C. rosea* PKS gene expression during fungal-fungal interactions may be influenced by the fungal prey. The mycotoxin deoxynivalenol produced by *Fusarium* spp. was reported to suppress NAGase gene expression in *T. atroviride*^43^, indicating that fungal prey species may have evolved effectors that interfere with the activity of their parasites. In this study, the *pks19* gene is specifically suppressed in *C. rosea* interacting with *F. graminearum*, but not with *B. cinerea*. This is interesting, as *pks19* is the core gene in a putative depudicin gene cluster in *C. rosea*, reported to be conserved among distantly related groups of fungi^44^. Depudicin was shown to be an inhibitor of histone deacetylases (HDACs)^31,45^ that together with histone acetyltransferases (HATs) influence expression of genes involved in various processes, including secondary metabolite production^46,47^, through chromatin modifications. Chromatin modification-mediated gene expression has previously been suggested in mycoparasitic *Trichoderma* spp. during *in vitro* interaction with the plant pathogenic fungus *R*. *solani*^41^.

However, the fact that all PKS genes in *C. rosea* are expressed under the standard laboratory conditions used in this study indicates a role for polyketide compounds in normal growth and development in *C. rosea*. Many PKS genes in *T. reesei* were also reported to be highly expressed during vegetative growth, suggesting diverse functional roles of polyketides^48^. The induction of 19 PKS genes during ageing and pigmentation of *C. rosea* also indicate that certain PKS genes encode enzymes involved in pigment biosynthesis, as shown for the *pks4* gene in *T. reesei*^12^. Induced expression of PKS genes during pigmentation and pigment secretion were shown for several different fungi including *B*. *cinerea*^49^, *F*. *graminearum*^7^, *F*. *fujikuroi*^39,40^, *M*. *purpureus*^50^ and *T. reesei*^41,51^. For instance, *C. rosea* is predicted to harbour orthologs of the sorbicillinoid family-type yellow pigment sorbicillin (*pks2* and *pks12*) gene cluster^52,53^, conserved and functionally active in several distantly related fungi such as *Acremonium chrysogenum*, *P. chrysogenum*, *T. reesei*, *Chaetomium globosum* and *Colletotrichum graminicola*^30,51^. In addition, *C*. *rosea* PKS1 shares similar protein domain organization (SAT-KS-AT-PT-ACP) and was shown to be phylogenetically close to the AptA PKS of *A. nidulans*^25^, known to be involved in biosynthesis of the anthraquinone-type yellow pigment asperthicin^54^. All three genes (*pks1*, *pks2* and *pks12*) were highly induced during yellow pigment secretion in the current study suggesting biosynthesis of sorbicillin-like and asperthicin-like compounds in *C. rosea*.

Based on their induction during growth in PDB and SNB media, during antagonistic interactions, but not during pigmentation, we selected *pks22* and *pks29* for functional characterization under the hypothesis that they encode PKS proteins that biosynthesize polyketide compounds with biotic activities. Deletion of *pks22* allowed us to identify several previously unknown secondary metabolites that were named Clonorosein A-D. By determining the structures of Clonorosein A and B using NMR and MS we showed that these compounds are methyl substituted octenoic or octanoic acid derivatives. The backbone of Clonorosein is likely to be assembled by PKS22, which is subsequently methylated on carbon atoms number 2, 4 and 6, possibly by the MT domain present in PKS22. Similar polyketides, i.e. fatty acid derivatives with methyl substitutions on every second carbon, have previously been described in the genus *Clonostachys*^34,55–57^. Taken together, deletion of *pks22* combined with the identification of Clonorosein A-D as methylated fatty acid derivatives further supports that PKS22 is a PKS that is involved in the biosynthesis of Clonorosein.

A bioactivity test further proved the antifungal activity of purified Clonorosein A and B against *B*. *cinerea* and *F*. *graminearum* that fits well with the increased *pks22* gene expression during interactions with the same fungi, suggesting a role of PKS22 in fungal-fungal interactions. The lack of observable phenotypic differences between Δ*pks22* and WT strains with regard to *in vitro* antagonism and *in planta* biocontrol may be explained by production of additional compounds or enzymes with antifungal activity by *C. rosea* that effectively compensate for the absence of Clonorosein.

Deletion of *pks29* resulted in strains with 50% reduced ability to produce a compound with the molecular formula C_15_H_28_O_3_, suggesting that PKS29 is involved in the biosynthesis of this compound. However, the fact that 50% of the compound is still produced by the Δ*pks22* strains suggests the involvement of an additional gene in the biosynthesis of compound 5. There is an example in the human pathogen *Aspergillus fumigatus* where two BGCs are involved in the biosynthesis of an endocrocin polyketide through two distinct routes^58^. PKS29 was in fact reported to be part of a phylogenetic group with several, closely related paralogous PKSs in *C. rosea*^25^, possibly involved in producing compound 5 or the derivate of compound 5 eluted at 74 s with the same m/z.

The significant increase in *B*. *cinerea* biomass when grown in Δ*pks29* culture filtrates suggests a role of PKS29 and compound 5 in *in vitro* antagonism. This is consistent with gene expression data where *pks29* showed the highest induction among all PKS genes in PDB medium. PKS29 is further required for full biocontrol ability of *C. rosea* against foot rot disease on barley caused by *F*. *graminearum*, again consistent with the induction of *pks29* expression in *C. rosea* during interaction with *B. cinerea* and *F. graminearum*.

In addition to their role in antagonism, deletion of *pks22* and *pks29* also resulted in several phenotypic effects related to growth and reproduction in *C*. *rosea*. This plausibly is related either with the physiological costs of secondary metabolite production which otherwise are used for growth and conidiation, or with disturbance in metabolite production machinery in Δ*pks22* and Δ*pks29* deletion strains that in turn prompted the alteration in growth and conidiation^59^. Furthermore, our data from phenotypic analysis where deletion strains showed different phenotypic effects on PDA and CZ, with the exception of Δ*pks22* that showed reduced growth rate on both media, suggested that the functions of PKS22 and PKS29 are culture medium dependent and confirm the gene expression pattern where expression of *pks22* or *pks29* was significantly different in PDB and CZ.

The involvement of PKSs in normal mycelial growth and development, production of sexual and asexual spores, and pigmentation have previously been shown in filamentous fungi. For example disruption of *GRS1* or *PKS2* inhibited mycelial growth, while disruption of *AUR1* and its homologue *Fsr1* affected perithecium pigmentation in *F*. *graminearum* and *F*. *verticillioides*^6,7^. Similarly, fluP disruption mutants in *A. parasiticus* had significant reduction in mycelial growth and sporulation^60^. In *T*. *reesei* and *Sordaria macrospora*, loss and over-expression of *pks4* resulted in disrupted sexual development^8,12^. Our results did not indicate any role of PKS22 or PKS29 in pigmentation, which is consistent with the lack of induction of either gene during pigmentation.

Given the large numbers of PKS genes in mycoparasitic fungi, surprisingly little is known about their functions in governing microbial interactions resulting in biological control of plant diseases. We here show that PKS gene expression in *C. rosea* is regulated both by nutrient conditions and by interactions with other fungi. Our results from antagonism assay using culture filtrate test showed that *pks29* is involved in antagonism against *B*. *cinerea*. Furthermore, we identified two previously unknown polyketide compounds from *C*. *rosea* with antifungal activity, Clonorosein A and B.

## Methods

### Fungal strains and culture condition

Cultures of *C. rosea* IK726 WT and deletion mutants derived from it, *A*. *alternata* TUCIM 3594, *B*. *cinerea* strain B05.10, *F*. *graminearum* strain PH1, and *R*. *solani* strain SA1were maintained on PDA (Sigma-Aldrich, St. Louis, MO) plates at 25 °C.

### Sequence analysis

The protein accession numbers of PKSs of *C. rosea* were retrieved from Karlsson *et al*.^25^, and their aa and nucleotide sequences were retrieved from GenBank at NCBI. The PKS protein domain architectures were analysed and annotated based on their aa sequence using simple modular architecture research tool (SMART)^61^ and conserved domain database (CDD)^62^. Antibiotic and secondary metabolite analysis shell (antiSMASH) version 4 web server http://antismash.secondarymetabolites.org was used to identify putative PKS BGCs in *C. rosea* genome^63,64^. The search was performed on annotated genome sequence following procedure described by the developer^63,64^. For each predicted ORF in clusters, a BlastP search was done using predicted aa sequences against *C*. *rosea* protein sequences to identify PKS gene part of the predicted biosynthetic gene cluster, and also to identify their predicted aa sequences.

### Analysis of *C. rosea* antagonism in different culture media

Five different culture media: CZ (Sigma-Aldrich, St. Louis, MO); PDB (Sigma-Aldrich, St. Louis, MO), ME (Duchefa Biochemie, Haarlem, Netherlands); SMS, and SNB with different carbon and nitrogen source and pH were used to study antagonistic behaviour of *C. rosea* under different nutritional conditions (Table S3). *C. rosea* conidia (1 × 10^7^conidia) isolated from a 2 week old plate were used to inoculate 1000 ml flasks containing 250 ml of culture medium. Flasks were incubated at 25 °C on a rotary shaker (100 rpm), and culture filtrates were obtained 4 dpi by removing fungal mycelia using vacuum filtration. Culture filtrates were passed through 0.45 µM cellulose acetate membrane syringe filters (Sarstedt Aktiengesellschaft & Co., Nümbrecht, Germany) to remove the mycelial debris before use. Harvested mycelia were washed with distilled water, frozen immediately in liquid nitrogen and stored in −70 °C for RNA extraction to use for gene expression analysis. To measure the mycelial biomass, a five mm agar plug of *A*. *alternata*, *B*. *cinerea*, *F*. *graminearum* or *R*. *solani* was inoculated into 50 ml flasks containing 10 ml of culture filtrate and were incubated at 25 °C on a rotary shaker with constant shaking. Fungus inoculated into respective fresh culture medium was used as control. Mycelial biomass was harvested 4 dpi using vacuum filtration, dried at 65 °C and weighed to determine the mycelial dry weight. The experiment was performed in five biological replicates.

### Quantitative reverse transcription polymerase chain reaction

For gene expression analysis in liquid CZ, PDB and SNB culture media, *C. rosea* mycelia were cultivated and harvested 4 dpi as described in the previous section. *C*. *rosea* inoculated into CZ medium was used as a control treatment. For gene expression analysis during dual culture interactions with *F*. *graminearum* and *B*. *cinerea*, *C. rosea* was grown and harvested as described previously^11,27^. Mycelium harvested at same stage from *C*. *rosea* confronted with *C*. *rosea* (Cr–Cr) was used as control treatment. For gene expression analysis during pigmentation, a 5 mm agar plug of *C. rosea* was inoculated on PDA medium covered with cellophane membrane for easy harvesting of fungal mycelia, and incubated at 25 °C in dark to avoid conidiation. Mycelia ware harvested 10 dpi as at this stage production of yellow pigment was apparent on bottom of agar plates. Four days old *C. rosea* culture plates, incubated at the same conditions were used as control treatment as no pigmentation was observed at this stage.

Harvested mycelia were flash frozen in liquid nitrogen, freeze-dried (VirTis Sp scientific, Warminster, PA) at −95 °C, and then homogenized into a fine powder using Precellys 24 lysis and homogenization (Bertin Technologies, France). Immediately after homogenization, total RNA was extracted using Qiagen RNasy plant mini kit (Qiagen, Hilden, Germany). For cDNA synthesis, 1000 ng of total RNA, after treating with DNase I (Fermentas, St-Leon-Rot, Germany), was reverse transcribed (RT) in a total volume of 20 µl using iScript™ cDNA Synthesis Kit (Bio-Rad, Hercules, CA). The transcript levels were quantified by quantitative polymerase chain reaction (RT-qPCR) using gene specific primer pairs (Table S4A) in a 20 µl reaction mix as described previously^65^. Primer amplification efficiency of each primer pair was determined by amplifying serial dilutions of *C. rosea* IK726 genomic DNA. Melt curve analysis was performed after the qPCR reactions to confirm that the signal was the result from a single product amplification. Cycle threshold (Ct) values were log transformed, mean centered and autoscaled as described previously^66^. Relative expression levels for the target gene in relation to actin and β-tubulin^11,27,65^ were calculated using the 2^−ΔΔCt^ method^67^. The gene expression experiments were performed in five biological replicates, and each replicate had two technical replicates.

### Construction of deletion vector, transformation and mutant validation

Genomic DNA was isolated following a CTAB-based protocol^68^. Dream Taq DNA polymerase (Thermo Fisher Scientific) was used for PCR amplification of ~1 kbp 5′ -flank and 3′ -flank regions of the *pks22* and *pks29* orfs from genomic DNA using primer PKS22ups F/PKS22ups R, PKS22ds F/PKS22ds R; and PKS29ups F/PKS29ups R, PKS29ds F/PKS29ds R, respectively (Table S4B). Gateway entry clones of the purified 5′-flank and 3′-flank PCR fragments were generated as described by the manufacturer (Invitrogen, Carlsbad, CA). The entry clone of hygromycin casette (hygB) constructed during our previous studies was used^69,70^. The deletion vector was constructed by performing the gateway LR recombination reaction as described previously^11,27^ and following manufacturer’s instructions (Invitrogen, Carlsbad, CA).

ATMT was performed based on a previous protocol for *C. rosea*^71^. Transformed strains were selected on plates containing hygromycin (200 μg/ml) and cefotaxime (300 mM). Putative transformants were tested for mitotic stability, and were purified by two rounds of single spore isolation^27,69^. Validation of homologous integration of the deletion cassette in putative transformants was performed using a PCR screening approach as described before^11,27,33,69,70^ using primers specific to the hygB cassette (Hyg F/Hyg R) in combination with primers specific to sequences flanking the deletion cassette (PKS22ko F/PKS22ko R or PKS29ko F/PKS29ko R) (Table S4B, Fig. S3A). Semi-quantitative RT-PCR analysis was conducted on WT and deletion strains using RevertAid premium reverse transcriptase (Fermentas, St.Leon-Rot, Germany) and primer pairs specific to *pks22* or *pks29* (Table S4B).

### Phenotypic analysis

All phenotypic analyses were performed using PDA and CZ media unless otherwise specified. 3 mm agar plug from the growing edge of *C. rosea* strains was inoculated onto PDA or CZ plates, incubated at 25 °C, and mycelial growth rate and colony morphology was recorded daily. For conidiation analysis, conidia were harvested from a 12 days old plate in 10 ml distilled water, and filtered through Miracloth to remove the mycelial debris. Conidial concentration was determined under the microscope using a bright line haemocytometer (Sigma-Aldrich, St. Louis, MO). Antagonistic behaviour of *C. rosea* WT and deletion strains was determined on PDA and CZ using an *in vitro* dual culture assay on solid medium, and a culture filtrate test in liquid culture medium. The *in vitro* plate confrontation assay was performed against *A*. *altarnata*, *B*. *cinerea*, *F*. *graminearum* or *R*. *solani* following procedures described previously^27^. Mycelial growth of *C. rosea* and the prey fungi was recorded daily. The culture filtrate test against *B*. *cinerea* or *F*. *graminearum* was performed as described previously^11,27^.

The biocontrol activity of *C. rosea* WT and deletion strains was assessed in an *in vivo* bioassay for *F*. *graminearum* foot rot disease on barley using a sand seedling test as described previously^11,27^. In brief, barley seeds were surface-sterilized with 2% NaOCl and air-dried on the benchtop of a laminar air-flow. The seeds were then coated with the conidial suspensions (1 × 10^8^ conidia/ml in water) of *C. rosea* WT or deletion strains for 30 minutes in a rotary shaker (125 rpm). Seeds incubated with sterile distilled water were used as a control. The seeds were then sown in pre-wetted sand in the 5 × 5 × 5 cm plastic pots (3 seeds/pot). For pathogen inoculation, a 5 mm agar plug of fresh *F*. *graminearum* mycelium was placed close to the seeds in the plastic pot. A PDA plug without *F*. *graminearum* was used as a control. The pots were then incubated in a growth chamber with a photoperiod of 12 h light (150 µmol m^2^ s^−1^ light intensity)/12 h dark, 70% ± 5% relative humidity, and 15 ± 1 °C^11,27^. Seedlings were harvested 3 weeks post-inoculation, and disease symptoms were scored on a 0 to 4 scale as described before^11,27^. The experiment was performed in five biological replicates with 15 plants per replicate.

### Secondary metabolite analysis

For secondary metabolite analysis, *C*. *rosea* WT and deletion strains were cultivated in 1000 ml flask containing 200 ml PDB medium for 15 days. Culture filtrates were collected by removing mycelial debris by centrifugation (13000 rpm for 5 min). Culture filtrates (injection volume 0.5 µL) were analyzed by UHPLC-MS on a reversed phase column (2.1 × 50 mm, 1.5 μm, Accucore Vanquish, Thermo Scientific, Waltham, MA, USA) using a gradient of acetonitrile (MeCN) in water, both with 0.2% formic acid (10–95% MeCN in 3 min, 95% MeCN for 1.2 min, at 0.9 mL min^−1^). The MS was operated in positive mode with scanning of *m/z* 50–1500, and the mass spectra were calibrated against sodium formate clusters. MS-MS was performed on the same instrument with 2 amu isolation width and 24 eV fragmentation energy.

For isolation and identification of secondary metabolites, cell-free culture supernatants (total volume 1000 mL) were fractionated on 4 × 10-g C-18 SPE columns. Following sample loading, each column was washed with 50 mL 25% MeCN in water and then eluted with 50 mL 95% MeCN in water. The combined 95% MeCN extract was dried under reduced pressure and re-dissolved in 4 mL 60% MeCN in water, and fractionated (4 × 1 mL injected) by gradient preparative reversed phase HPLC (21.2 × 100 mm, 5 μm, Hypersil Gold, Thermo Scientific, Waltham, MA, USA) using a gradient of MeCN in water (10–95% MeCN in 10 min, followed by 10 min at 95% MeCN, at 10 mL min^−1^). Fractions were analyzed by UHPLC-MS as above, and fractions containing the different target compounds, were pooled and dried under reduced pressure. The dried fractions were then subjected to isocratic reversed phase HPLC (fraction region A: 35% MeCN in water, 0.2% formic acid, 10 mL min^−1^; fraction region B: 47.5% MeCN in water, 0.2% formic acid, 10 mL min^−1^; column as above), and fractions containing the selected compounds, as indicated by UHPLC-MS (as above), were collected and dried in a vacuum centrifuge, and their structures were studied by NMR and MS.

^1^H and ^13^C NMR data were acquired in CDCl_3_ on a Bruker Avance III 600 MHz NMR spectrometer (Bruker Biospin GmBH, Rheinstetten, Germany) equipped with a 5-mm cryo-probe (^1^H, ^13^C, ^15^N, ^31^P). Standard pulse sequences supplied by Bruker were used for acquisition of 1D ^1^H, COSY, TOCSY, DEPT-HSQC, HMBC, and ROESY NMR data. Chemical shifts were determined relative to internal chloroform (δ_C_ 77.23; δ_H_ 7.27). UHPLC-MS and UHPLC-MS-MS were performed on an Agilent 1290 Infinity II UHPLC (Agilent, Palo Alto, CA, USA) connected to a Bruker maXis Impact ESI Q-TOF MS (Bruker Daltonic GmbH., Bremen, Germany). Preparative HPLC was run on a Gilson 306/306 pump system (Gilson Inc., Middleton, WI, USA) with a Gilson 119 UV/VIS detector monitoring at 210 nm. Fractions were collected with a Gilson 204 fraction collector and collection was done in polypropylene 2.2 ml square well plates (VWR, Radnor, PA, USA). MeCN of HPLC gradient grade (Sigma-Aldrich, St. Louis, MO, USA) and deionized filtered water (Millipore, Billerica, MA, USA) were used for preparation of mobile phases. Polarimetry was performed on a Perkin Elmer 341 polarimeter (λ 589 nm, path length 10.0 cm, 20 °C) with compounds dissolved in methanol.

### Biological activity assay

Pure compounds dissolved in MeOH were transferred into 96-well microtiter plates, and solvents were evaporated in a fume hood at room temperature. The bioactivity of the compounds was tested at eight concentrations, 1, 2, 4, 8, 16, 32, 64, and 128 µg/ml. A suspension of 5 × 10^3^ *B*. *cinerea* or *F*. *graminearum* spores in 100 µl of half PDB was added in each well, and plates were incubated at 25 °C temperature in dark. To evaluate the effects of traces of residual methanol that may present in well, five concentration of methanol corresponding to 1% 2%, 4%, 8%, and 16% was tested for its effect on conidial germination and germ tube growth of *B*. *cinerea* or *F*. *graminearum*. A spore suspension in half PDB only was used as control treatment. After 8 hour of incubation, photographs of the germinating conidia were recorded in a Leica DM5500M Microscope at 10 X or 20 X magnification using a Leica DFC360FX digital camera (Wetzlar, Germany). Frequency of conidial germination was determined by counting the number of germinating and non-germinating conidia, while length of the germ tube was measured using ImageJ software^72^. Conidia were counted as germinated if they had germ tubes at least as long as the length of the conidia. Abnormal growth and morphological differences such as lysis of the tips were also noted. Two hundred to three hundred conidia were counted and length of their germ tube was measured for each replicate. The experiment was performed in three biological replicates.

### Statistical analysis

All the statistical analyses in this study were performed using Minitab 16 statistical software or Statistica version 10 (Stat-Soft, Tulsa, OK). Significant differences among the treatments were tested using one-way analysis of variance (ANOVA) while pairwise comparisons were made using Fisher’s exact test or Tukey-Kramer test with 95% level of confidence.

## Electronic supplementary material

Supplementary Figures

Supplementary Table S1

Supplementary Table S2
