# Supplementary material for: Functional analysis of polyketide synthase genes in the biocontrol fungus Clonostachys rosea
Source: Sci Rep. 2018 Oct 9;8:15009. doi: 10.1038/s41598-018-33391-1 (PMC6177402; doi:10.1038/s41598-018-33391-1)
Supplement: Supplementary file 1 — Supplementary Figures [file 41598_2018_33391_MOESM1_ESM.pdf]

Functional analysis of polyketide synthase genes in the biocontrol fungus *Clonostachys rosea*

Umma Fatema[1§], Anders Broberg[2], Dan Funck Jensen[1], Magnus Karlsson[1], and Mukesh Dubey[1*]

[1]Department of Forest Mycology and Plant Pathology, Uppsala Biocenter, Swedish University of Agricultural Sciences, P.O. Box 7026, SE-75007, Uppsala, Sweden.

[2]Department of Molecular Science, Uppsala BioCenter, Swedish University of Agricultural Sciences, Box 7015, SE-75007 Uppsala, Sweden.

[§]Current address: Umma Fatema, Department of Plant and Soil Sciences, 412 Plant Science Building 1405 Veterans Drive, University of Kentucky, Lexington, KY 40546-0312, USA

*****mukesh.dubey@slu.se.

Table S3: Culture medium composition used to analyse *C. rosea* antagonism, and PKS gene expression

Name	Composition (g/L); pH
Czapek-Dox (CZ)	Sucrose: 30, $NaNO_3$: 2, K_2HPO_4: 1, $MgSO_4$: 0.5, KCL: 0.5, $FeSO_4$: 0.01; pH 7.3 ± 0.2
Malt extract (ME)	3% (w/v) malt extract; pH 5.5 ± 0.3
Potato dextrose broth (PDB)	Potato dextrose broth, 24g (potato starch 4 g, dextrose 20 g); pH 5.1 ± 0.2
Synthetic minimal salt (SMS)	Glucose: 10, KH_2PO_4: 2, $(NH_4)_2SO_4$: 1.4, $MgSO_4.7H_2O$: 0.3, $CaCl_2.2H_2O$: 0.3, $FeSO_4.7H_2O$: 0.005, $ZnSO_4.7H_2O$: 0.002, $MnSO_4.H_2O$: 0.002; pH 5.1
Synthetic nutrient agar (SNB)	Glucose: 0.2, sucrose: 0.2, KH_2PO_4: 1, KNO_3: 1, $MgSO_4.7H_2O$: 0.5, KCl: 0.5; pH 4.62

Table S4A: List of primers used in gene expression analysis.

Primer name	Forward primer sequence	Reverse primer sequence	Target	Amplicon size (bp)
PKS1	tggggttttactgggtgtgctc	gatacccagcccagtaaccacag	*pks1*	159
PKS2	tctcggctactggtgctctaagg	ttggaagaacatgggcgtagtg	*pks2*	205
PKS3	gtggtgggggaactttttgaac	gcagccataatctaggggttcag	*pks3*	183
PKS4	gctgcacctttccactcgttc	gtaaactgctgctgaaacggactc	*pks4*	129
PKS5	ctccggcctgctaaagttgttg	gtaagccgagtgccatcatagagc	*pks5*	186
PKS6	tccccaacgaacgcatctttag	cgttcaactgagcgtccttctttc	*pks6*	188
PKS7	cattcggggcacagacacaaac	gcttcgatgtagcgtgtttggtc	*pks7*	137
PKS8	caccgcatgttcatcctcactc	acgcccattctcgctgtattc	*pks8*	140
PKS9	aataaaccacggcccttccttc	accatcccacttgctatccgtc	*pks9*	197
PKS10	acatctcccgtccgtttcagtg	gcaaggctcagcagggtaacag	*pks10*	187
PKS11	catgggggattcaagcagagtc	gacgcatgcaacacaaacacg	*pks11*	175
PK12	caaatggcaggggagtagacg	gaaactgagcgggagaaggaac	*pks12*	162
PKS13	atggggaaggctatgggctatg	ctattcaaggccgccaccatc	*pks13*	202
PKS14	gaggctggctcgaatcaagac	tgcatagtcaccagcgtccag	*pks14*	192
PKS15	gttgcagggctgatcaaaggag	ggccagtccattaatcgtgtagg	*pks15*	136
PKS16	aggcagcagtcattcctatcgtg	gcttctcaagactgccgactgtg	*pks16*	200
PKS17	gaatgccatggaacaggaacac	gcctacgcttggtttcacactg	*pks17*	131
PKS18	cgttgatgcgttaagcgagattg	ccatggctccagctttctttacc	*pks18*	169
PKS19	gcaatgaaccaacagcgaacc	cctcttgtcggtgggataatgc	*pks19*	151
PKS20	gttcccctttgcctgctacattg	gatgctgaactcccaccactgac	*pks20*	201
PKS21	tctgcccaagctcctcgtattc	gatctccataacatgcggctgc	*pks21*	164
PKS22	tggaaagagctgcagacgagag	gagcgacaagggctgatgaac	*pks22*	156
PKS23	gatgtctttgtcgccagcctc	ctccgagacatccgtaaaggtg	*pks23*	191
PKS24	aaacccgcttcatcaacgacac	cgaggcgtcaaactgggataga	*pks24*	124
PKS25	gccaatgatgatgcggtgaac	caaattcagcgggtccatctc	*pks25*	200
PKS26	gcactggtacgcagattggagac	gatggccgatgtttgctttgatag	*pks26*	119
PKS27	tgcacgcagcaggtctcaac	gcagaactcatcgggcacaac	*pks27*	202
PKS28	gagacgctccatccaaacatcc	tggtcgccgttctcaatcactac	*pks28*	151
PKS29	tggtgggaattcagcatctcg	gaatcgcggtccgtagttttg	*pks29*	197
PKS30	ggtatgaaaagcccgcaatcc	ggctgtgtactttgggatgacg	*pks30*	182
PKS31	catcatgatgggggctacagg	cagcgtcgcctaaagcaatg	*pks31*	186
PKS32	atccttcgagcgtggggtatc	acccttcctgctgggtcattc	*pks32*	195

Table S4B: List of primers used to construct gene deletion cassette, and in mutant's validation. attB and attBr sequences used for gateway cloning are underlined.

Primer name		Sequence 5´-3´	Target
PKS22ups	F	ggggacaactttgtatagaaaagttggacccgcaccaacttgagactat	upstream region of *pks22* for deletion cassette
	R	ggggactgcttttttgtacaaacttgrccctacccacaatcaaccaactt	
PKS22ds	F	ggggacagctttcttgtacaaagtggcttccacaggagaggggacttac	downstream region of *pks22* for deletion cassette
	R	ggggacaactttgtataataaagttgacaatcagatccaccagcctacc	
PKS22ko	F	actttgagcctggcatcgtatc	100-200 bp upstream (forward primer) and downstream (reverse primer) to *pks22* deletion cassette
	R	tgaggtgattgggagcgtagat	
PKS29ups	F	ggggacaactttgtatagaaaagttgtgtgtccatgaggtgtgaagtcc	upstream region of *pks29* for deletion cassette
	R	ggggactgcttttttgtacaaacttgcgtctcggtttctgtgttgttttg	
PKS29ds	F	ggggacagctttcttgtacaaagtgggcgagttgaagatgacgaaggat	downstream region of *pks29* for deletion cassette
	R	ggggacaactttgtataataaagttgataaccggatgcaaaacagtcttc	
PKS29ko	F	ggaactcggcttcttacaactgac	100-200 bp upstream (forward primer) and downstream (reverse primer) to *pks29* deletion cassette
	R	ctttggaaccactgccttgactt	
Hyg	F	gcgcgcaattaaccctcac	Hygromycin (*hygB*) cassette
	R	gaattgcgcgtacagaactcc	

Figure S1

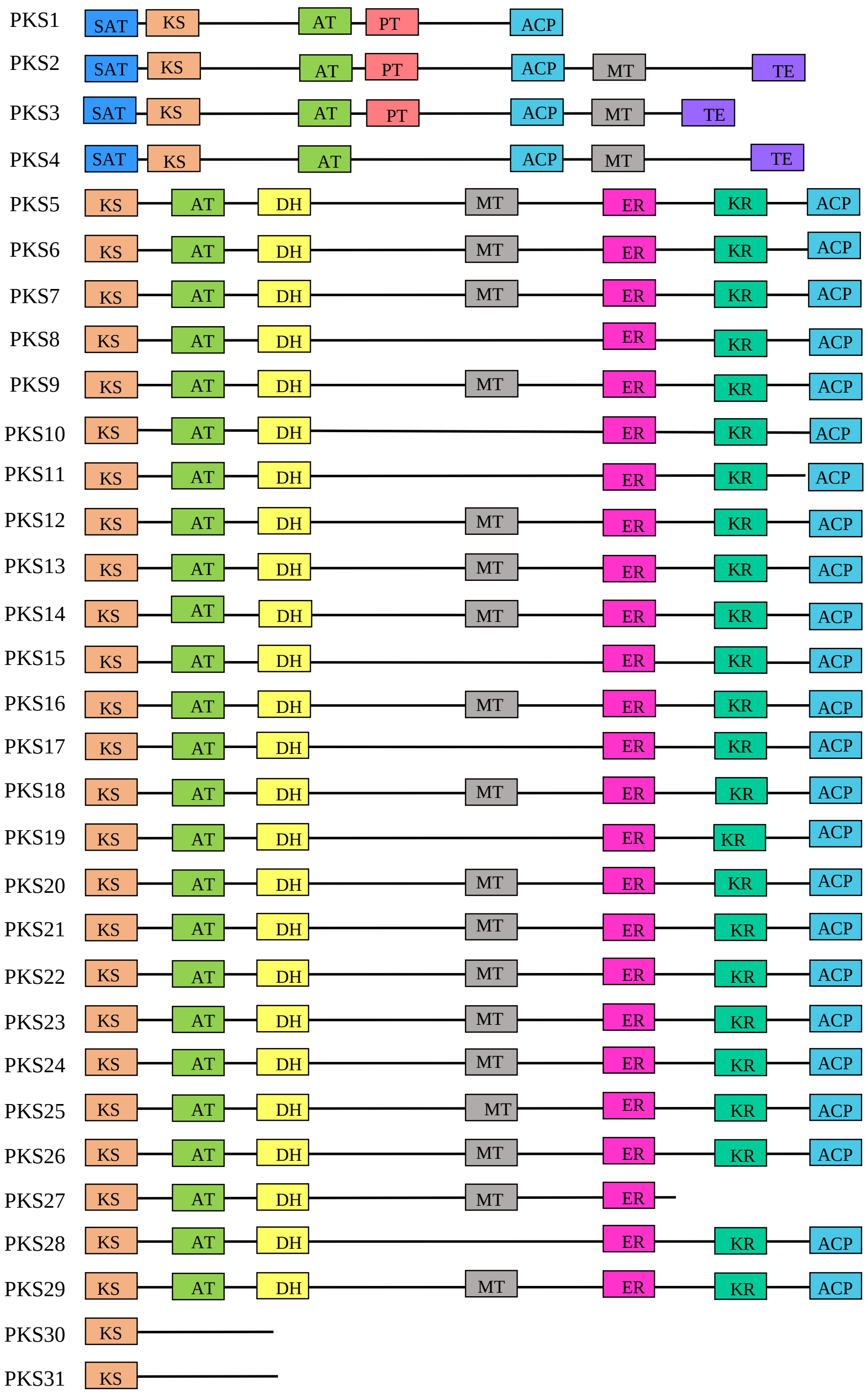

ACP = Acyl carrier protein
AT = Acyl transferase
DH = Dehydratase
ER = Enoyl reductase
KR = Ketoreductase
KS = Ketoacyl synthase
MT = Methyltransferase
PT = Product template
SAT = Starter unit acyltransferase
TE = Thioesterase

Figure S1: Domains predicted to be present in *Clonostachys rosea* PKSs

E-values for domains in each *C. rosea* PKS. Simple Modular Architecture Research Tool (SMART) and Conserved Domain Database (CDD) was used to predict protein domains in each PKS protein. E-values are shown for each domain predicted**.**

	SAT	KS	AT	PT	DH	ER	KR	ACP	MT	TE
**PKS1**	2.7e-46	1.7e-69	1.13e-72	1.5e-9				9.8e-9		
**PKS2**	6e-10	7.9e-72	4.91e-59	1.02e-18				5.9e-9	1.4e-9	4.5e-18
**PKS3**	9.1e-4	2.2e-72	9e-21	3.5e-16				6.1e-14	2.7e-13	2.8e-13
**PKS4**	3.3e-10	4.2e-70	1.24e-55					1.9e-9	1.9e-07	1.7e-22
**PKS5**		9.34e-188	6.46e-109		1.32e-18	2.84e-137	2.33e-42	1.7e-06	1e-18	
**PKS6**		6.41e-186	3.72e-91		1.1e-20	9.94e-151	7.01e-46	1.5e-05	2.8e-10	
**PKS7**		1.39e-195	5.81e-102		8.8e-38	3.54e-147	7.2e-34	5.7e-11	1.4e-09	
**PKS8**		7.4e-63	1.49e-90		7.01e-14	1.76e-56	1.11e-43	5.7e-05		
**PKS9**		1.2e-185	4.75e-107		2.16e-14	2.45e-173	1.22e-36	5.9e-8	1.7e-08	
**PKS10**		3.3e-60	4.42e-84		3.12e-20	5.32e-119	1.38e-30	9.62e-09		
**PKS11**		1.18e-184	4.75e-66		1.67e-11	2.22e-130	3.23e-36	2.33e-04		
**PKS12**		1.33e-192	1.98e-104		1.75e-23	3.62e-112	1.18e-38	5.3e-05	2.1e-11	
**PKS13**		2.91e-198	1.62e-91		3.56e-15	1.32e-137	5.44e-43	7.6e-06	2e-18	
**PKS14**		5.2e-66	1.33e-96		1.2e-30	1.83e-149	1.35e-38	4e-7	8e-07	
**PKS15**		9.98e-206	5.8e-70		6.89e-22	3.98e-13	1.99e-36	6.91e-07		
**PKS16**		8.26e-189	2.49e-101		2.07e-16	2.9e-143	3.92e-35	2.6e-7	8.5e-05	
**PKS17**		3.59e-198	5.43e-93		4.24e-22	6.46e-36	1e-32	3.5e-05		
**PKS18**		1e-56	3.26e-96		4.94e-32	1.47e-162	9.92e-46	1.1e-05	5.9e-11	
**PKS19**		2.5e-66	9.68e-90		3.72e-18	1.88e-37	1.32e-41	1.62e-08		
**PKS20**		6.3e-60	3.09e-106		4.57e-27	4.64e-110	2.32e-37	6.4e-7	9e-08	
**PKS21**		2.23e-185	2.19e-97		9e-43	6.86e-81	4.36e-33	6.3e-8	3.8e-10	
**PKS22**		9.5e-58	4.21e-99		3.72e-27	2.04e-161	2.83e-41	4.5e-10	1.7e-7	
**PKS23**		6.2e-64	3.1e-88		2.55e-25	1.46e-98	2.95e-21	4.3e-7	6.3e-9	
**PKS24**		6.3e-31	2.25e-99		3.13e-34	4.9e-7	1.2e-14	2.5e-165	6.85e-49	
**PKS25**		5.8e-63	1.45e-93		5.3e-27	1.99e-150	2.32e-37	1.8e-9	1.3e-12	
**PKS26**		3.6e-42	3.95e-104		3.53e-10	3.44e-145	3.06e-37	1.4e-06	1.2e-13	
**PKS27**		3e-52	4.73e-93		3.44e-22	7.44e-100			2.8e-17	
**PKS28**		5.5e-51	1.76e-78		6.7e-41	2.39e-144	4.26e-36	1.4e-05		
**PKS29**		5e-24	1.31e-68		9.01e-26	2.52e-111	9.12e-45	6.4e-06	7.5e-17	
**PKS30**		4.2e-20								
**PKS31**		1.8e-58								

Figure S2

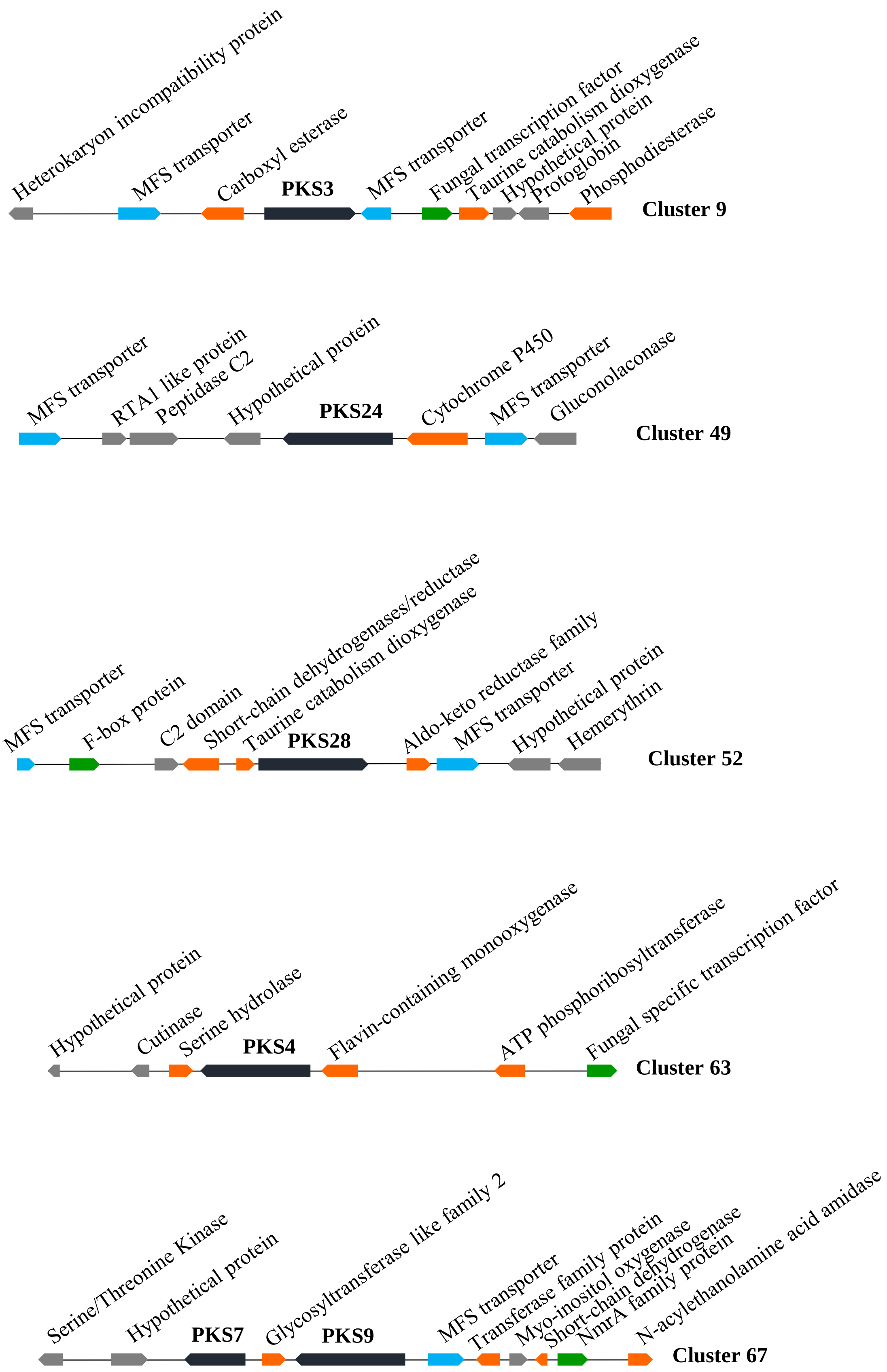
Heterokaryon incompatibility protein
MFS transporter
Carboxyl esterase
PKS3
MFS transporter
Fungal transcription factor
Taurine catabolism dioxygenase
Hypothetical protein
Protoglobin
Phosphodiesterase
Cluster 9
MFS transporter
RTA1 like protein
Peptidase C2
Hypothetical protein
PKS24
Cytochrome P450
MFS transporter
Gluconolaconase
Cluster 49
MFS transporter
F-box protein
C2 domain
Short-chain dehydrogenases/reductase
Taurine catabolism dioxygenase
PKS28
Aldo-keto reductase family
MFS transporter
Hypothetical protein
Hemerythrin
Cluster 52
Hypothetical protein
Cutinase
Serine hydrolase
PKS4
Flavin-containing monooxygenase
ATP phosphoribosyltransferase
Fungal specific transcription factor
Cluster 63
Serine/Threonine Kinase
Hypothetical protein
PKS7
Glycosyltransferase like family 2
PKS9
MFS transporter
Transferase family protein
Myo-inositol oxygenase
Short-chain dehydrogenase
NmrA family protein
N-acylethanolamine acid amidase
Cluster 67

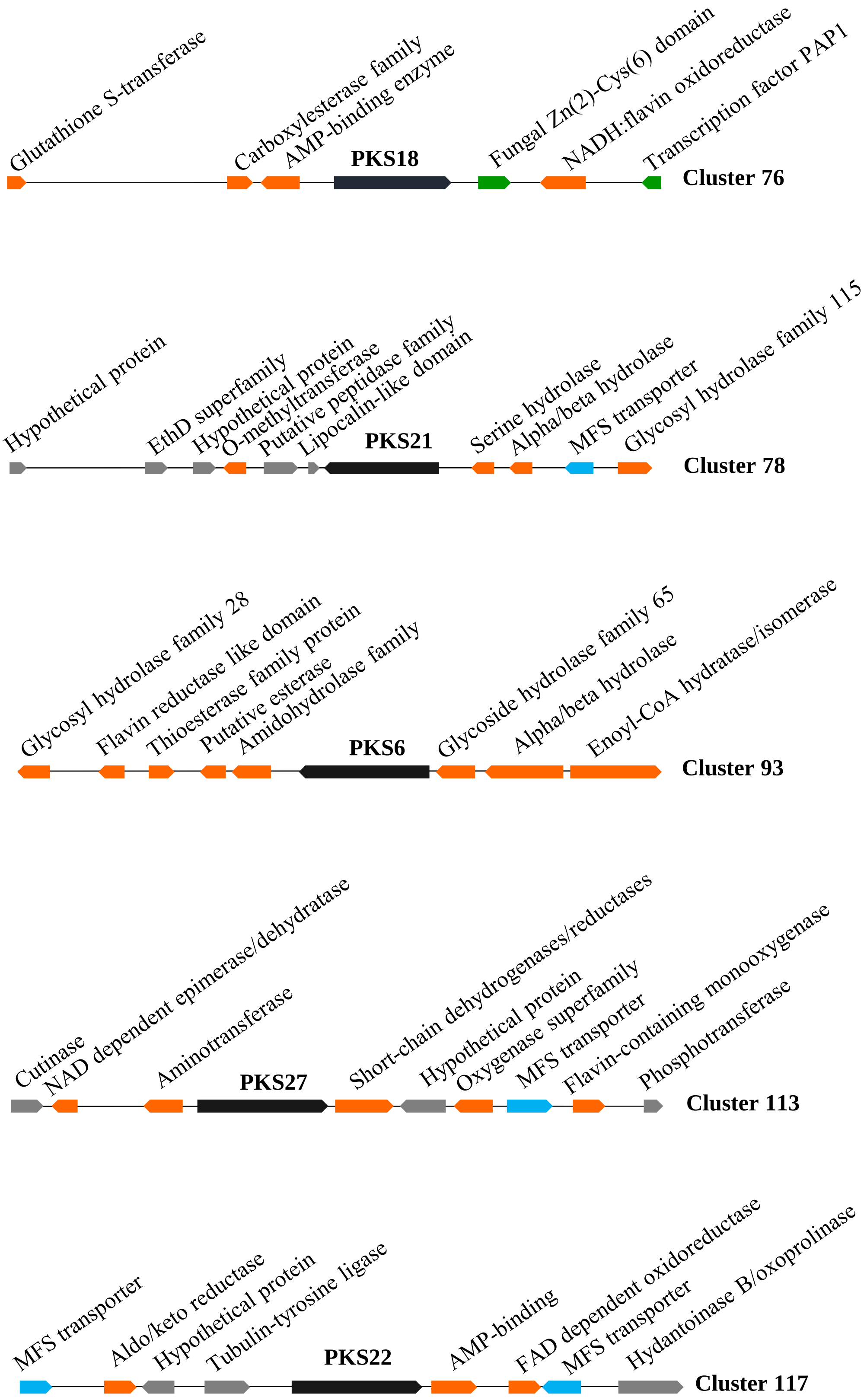
Glutathione S-transferase
Carboxylesterase family
AMP-binding enzyme
PKS18
Fungal Zn(2)-Cys(6) domain
NADH:flavin oxidoreductase
Transcription factor PAP1
Cluster 76
Hypothetical protein
EthD superfamily
Hypothetical protein
O-methyltransferase
Putative peptidase family
Lipocalin-like domain
PKS21
Serine hydrolase
Alpha/beta hydrolase
MFS transporter
Glycosyl hydrolase family 115
Cluster 78
Glycosyl hydrolase family 28
Flavin reductase like domain
Thioesterase family protein
Putative esterase
Amidohydrolase family
PKS6
Glycoside hydrolase family 65
Alpha/beta hydrolase
Enoyl-CoA hydratase/isomerase
Cluster 93
Cutinase
NAD dependent epimerase/dehydratase
Aminotransferase
PKS27
Short-chain dehydrogenases/reductases
Hypothetical protein
Oxygenase superfamily
MFS transporter
Flavin-containing monooxygenase
Phosphotransferase
Cluster 113
MFS transporter
Aldo/keto reductase
Hypothetical protein
Tubulin-tyrosine ligase
PKS22
AMP-binding
FAD dependent oxidoreductase
MFS transporter
Hydantoinase B/oxoprolinase
Cluster 117

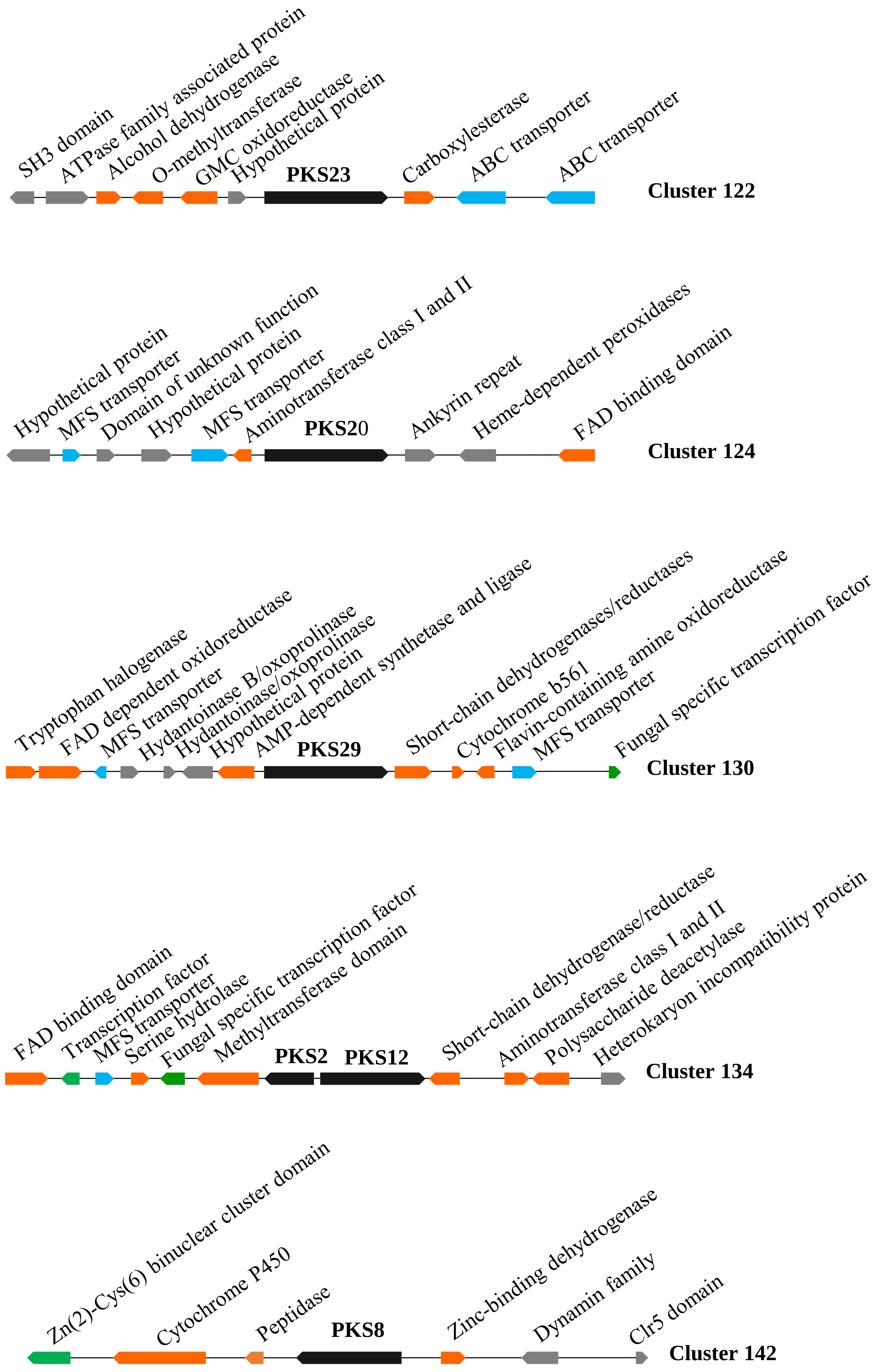
SH3 domain
ATPase family associated protein
Alcohol dehydrogenase
O-methyltransferase
GMC oxidoreductase
Hypothetical protein
PKS23
Carboxylesterase
ABC transporter
ABC transporter
Cluster 122
Hypothetical protein
MFS transporter
Domain of unknown function
Hypothetical protein
MFS transporter
Aminotransferase class I and II
PKS20
Ankyrin repeat
Heme-dependent peroxidases
FAD binding domain
Cluster 124
Tryptophan halogenase
FAD dependent oxidoreductase
MFS transporter
Hydantoinase B/oxoprolinase
Hydantoinase/oxoprolinase
Hypothetical protein
AMP-dependent synthetase and ligase
PKS29
Short-chain dehydrogenases/reductases
Cytochrome b561
Flavin-containing amine oxidoreductase
MFS transporter
Fungal specific transcription factor
Cluster 130
FAD binding domain
Transcription factor
MFS transporter
Serine hydrolase
Fungal specific transcription factor
Methyltransferase domain
PKS2
PKS12
Short-chain dehydrogenase/reductase
Aminotransferase class I and II
Polysaccharide deacetylase
Heterokaryon incompatibility protein
Cluster 134
Zn(2)-Cys(6) binuclear cluster domain
Cytochrome P450
Peptidase
PKS8
Zinc-binding dehydrogenase
Dynamin family
Clr5 domain
Cluster 142

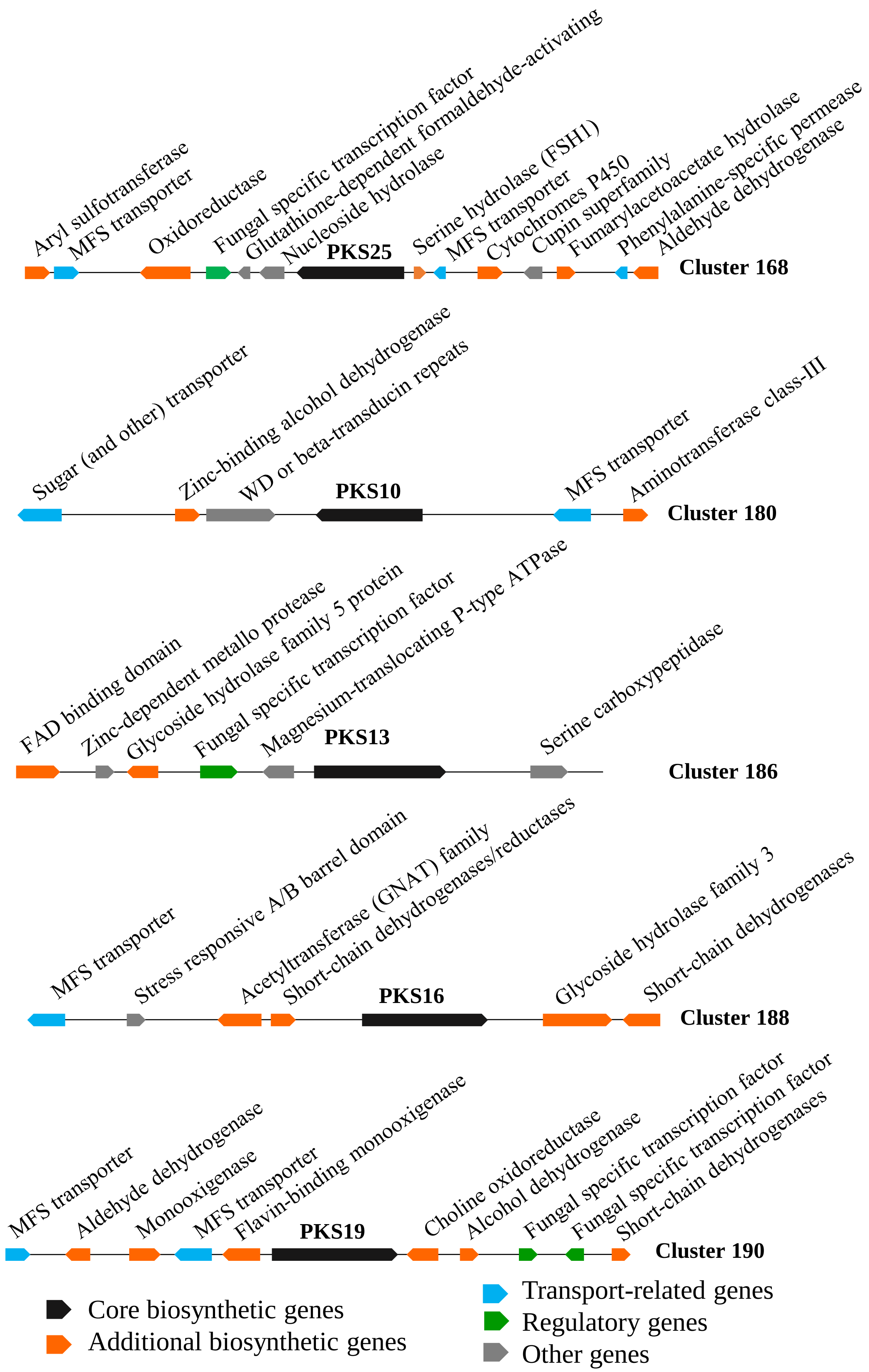

Figure S2: Schematic representations of the putative polyketide biosynthetic gene clusters in *Clonostachys rosea*. Genes with putative functions (indicated on top of each gene) of the corresponding proteins as determined by secondary metabolite cluster of orthologous groups (smCOG) and BLAST search are shown. (For more detail see Table S2).

Figure S3

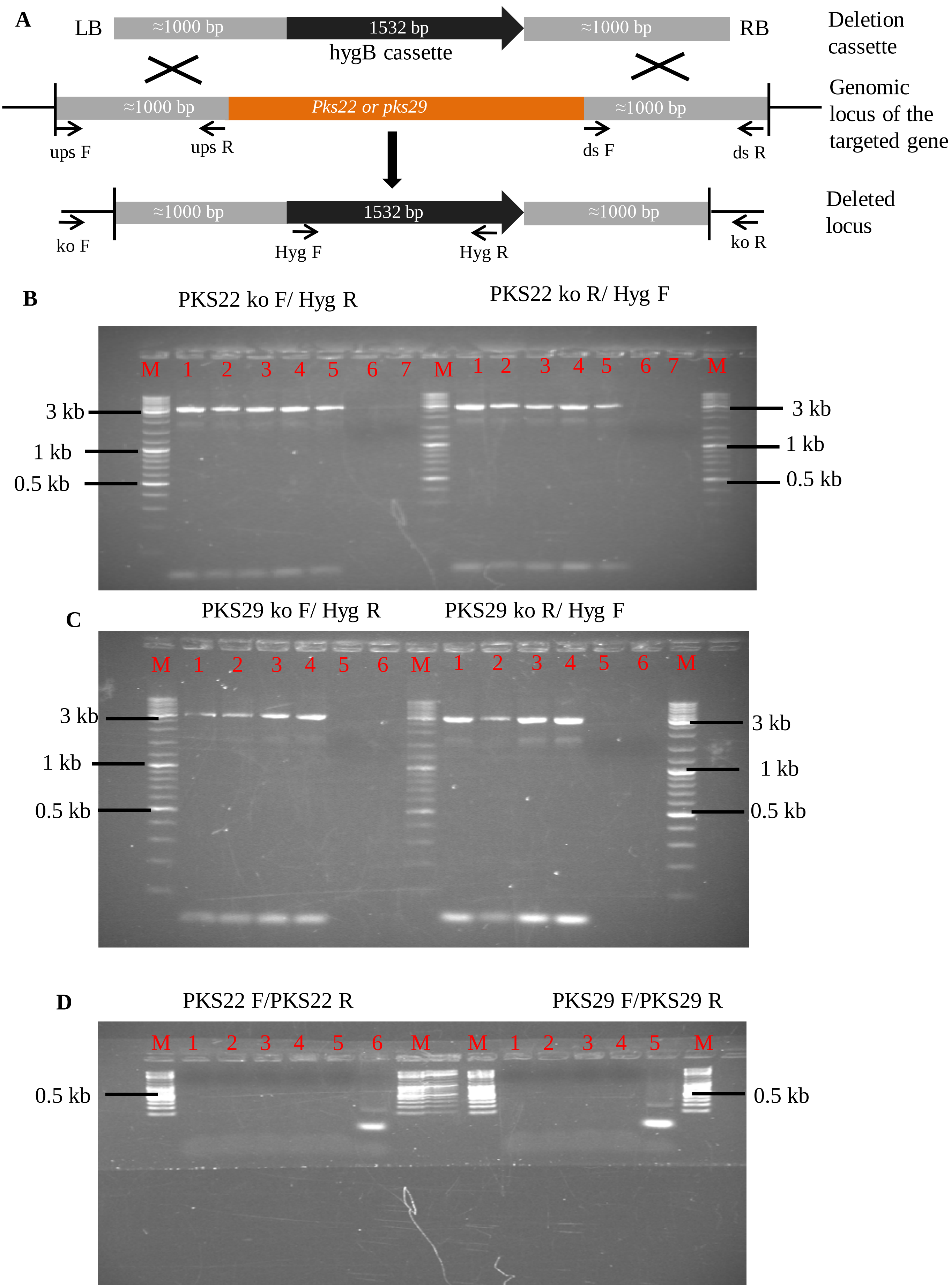
A
LB
≈1000 bp
1532 bp
≈1000 bp
RB
hygB cassette
Deletion cassette
≈1000 bp
Pks22 or pks29
≈1000 bp
Genomic locus of the targeted gene
ups F
ups R
ds F
ds R
≈1000 bp
1532 bp
≈1000 bp
Deleted locus
ko F
Hyg F
Hyg R
ko R
B
PKS22 ko F/ Hyg R
PKS22 ko R/ Hyg F
M 1 2 3 4 5 6 7 M 1 2 3 4 5 6 7 M
3 kb
1 kb
0.5 kb
C
PKS29 ko F/ Hyg R
PKS29 ko R/ Hyg F
M 1 2 3 4 5 6 M 1 2 3 4 5 6 M
3 kb
1 kb
0.5 kb
D
PKS22 F/PKS22 R
PKS29 F/PKS29 R
M 1 2 3 4 5 6 M M 1 2 3 4 5 M
0.5 kb

Figure S3: Schematic representation of deletion cassettes, and validation of mutant strains using PCR and RT-PCR.

A: Organisation of *pks22 or pks29* locus in WT and mutant strains of *C. rosea*. The coding region of respective gene was replaced by hygB cassette by homologous recombination resulting in generation of deletion strains. The arrow heads indicate the location of primers used to construct the deletion cassette and analysis of mutants using PCR. Abbreviations: LB, left border; RB, right border.

B: PCR verification of Δ*pks22* strains using primers located in the hygB cassette (Hyg F / Hyg R) in combination with primers located upstream and downstream from the deletion cassette (PKS22ko F / PKS22ko R). A PCR product of ~2.9 kb using primers PKS22ko F / Hyg R, and PKS22ko R / Hyg F were expected from a correct gene replacement. M, gene ruler DNA ladder mix; 1-5, independent Δ*pks22* mutants; 6, WT strain; 7, negative control. Primer combinations used for PCR and RT-PCR are given above the images.

C: PCR verification of Δ*pks29* using primers located in the hygB cassette (Hyg F / Hyg R) in combination with primers located upstream and downstream from the deletion cassette (PKS29ko F / PKS29ko R). A PCR product of ~2.9 kb using primers PKS29ko F / Hyg R, and PKS29ko R / Hyg F were expected from a correct gene replacement. M, gene ruler DNA ladder mix; 1-4, independent Δ*pks29* mutants; 5, WT strain; 6, negative control. Primer combinations used for PCR and RT-PCR are given above the images.

D: RT-PCR analysis of *pks22* gene expression in WT and deletion strains using *pks22* gene specific primers. A PCR product of 156 bp was expected from WT. M, gene ruler DNA ladder mix; 1-5, independent Δ*pks22* mutants; 6, WT strain Primer combinations used for RT-PCR are given above the images.
RT-PCR analysis of *pks29* gene expression in WT and deletion strains using *pks29* specific primers. A PCR product of 197 bp was expected from WT. M, gene ruler DNA ladder mix; 1-4, independent Δ*pks29* mutants; 5, WT strain. Primer combinations used for PCR are given above the images.
